# Preserved mitochondrial respiration in presence of oxidative stress and reduced mitochondrial mass after 10‐day bed rest in older adults

**DOI:** 10.1113/JP291588

**Published:** 2026-09-11

**Authors:** Evgeniia Motanova, Lucrezia Zuccarelli, Evgenii Lysenko, Stefano Amoretti, Marco Pirazzini, Ornella Rossetto, Lorenza Brocca, Roberto Bottinelli, Maria Antonietta Pellegrino, Giovanni Baldassarre, Clarissa Gissi, Giovanna Lippe, Mladen Gasparini, Boštjan Šimunic, Rado Pišot, Bruno Grassi, Marco V. Narici

**Affiliations:** ^1^ Department of Biomedical Sciences University of Padua Padua Italy; ^2^ Venetian Institute of Molecular Medicine Padua Italy; ^3^ Department of Medicine University of Udine Udine Italy; ^4^ Department of Biology University of Padua Padua Italy; ^5^ CIR‐MYO Myology Center University of Padua Padua Italy; ^6^ Institute of Neuroscience National Research Council Padua Italy; ^7^ Department of Molecular Medicine University of Pavia Pavia Italy; ^8^ Izola General Hospital Izola Slovenia; ^9^ Science and Research Center Koper Institute for Kinesiology Research Koper Slovenia

**Keywords:** inactivity, mitochondria, mitochondrial dynamics, oxidative metabolism, OXPHOS, ROS

## Abstract

**Abstract:**

Ageing affects mitochondrial integrity in skeletal muscle, and physical inactivity may further exacerbate these changes. Although mitochondrial alterations are documented in ageing and disuse independently, how disuse impacts the mitochondrial phenotype in older populations remains unclear. This work aimed to characterise how physical inactivity impacts mitochondrial function, morphology and gene expression in the skeletal muscle of older adults.

Ten healthy older men (65+ years) underwent 10 days of bed rest. Skeletal muscle biopsies were collected before and after bed rest to assess mitochondrial respiration (high‐resolution respirometry), H_2_O_2_ emission, mitochondrial protein expression, morphology and volume density (electron microscopy) and transcriptomic profile.

Ten days of inactivity increased mitochondrial reactive oxygen species (ROS) emission under non‐phosphorylating conditions but did not impair oxidative phosphorylation (OXPHOS) capacity, indicating preserved respiratory efficiency. Consistently, mitochondrial respiratory complex and supercomplex protein abundance were unchanged. Mitochondrial mass decreased, as shown by reduced mitochondrial volume density. Reduced dynamin‐like protein 1 (DRP1) phosphorylation at serine 637 was observed, whereas other mitochondrial fission and fusion protein levels remained unchanged. Mitochondrial morphology remained unaltered. Transcriptomic analysis revealed >3000 differentially expressed genes, characterised by downregulation of oxidative phosphorylation genes alongside altered mitophagy, antioxidant and oxidoreductase pathways.

In summary, 10‐day bed rest increased mitochondrial ROS emission and reduced mitochondrial mass in older skeletal muscle despite preserved respiratory function, indicating that elevated ROS production occurs upstream of respiratory dysfunction and is potentially linked to impaired antioxidant defence and ROS clearance.

These findings suggest that preserving redox balance during inactivity may be a key strategy to maintain muscle health and functional independence in ageing populations.

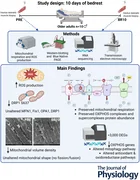

**Key points:**

- The impact of short‐term physical inactivity on mitochondrial function within the context of ageing remains poorly defined. This study examined the impact of 10‐day bed rest on skeletal muscle mitochondrial function, morphology and gene expression in older adults.
- Short‐term inactivity increased mitochondrial ROS production, accompanied by a dysregulation of antioxidant and oxidoreductase genes, indicating a reduced capacity for ROS clearance.
- Mitochondrial respiration was preserved under both submaximal and maximal stimulation. When normalised to mitochondrial content (citrate synthase activity), respiratory capacity increased, suggesting improved intrinsic efficiency.
- Mitochondrial mass was reduced, supported by decreased mitochondrial volume density assessed morphologically. Transcriptomic alterations in the mitophagy pathway suggest a potential role of altered mitochondrial degradation in this reduction.
- These findings indicate a transient compensatory response of ageing mitochondria to short‐term disuse, suggesting that functional impairments are likely driven by cardiovascular and microvascular factors rather than mitochondrial respiration itself.

## Graphical abstract

**Abstract figure legend** Ten older healthy male adults underwent 10 days of bed rest. *Vastus lateralis* muscle biopsies were collected at baseline (PRE) and after 10 days of bed rest (BR10), and mitochondrial adaptations were assessed using an integrative approach combining respirometry, protein analyses, RNA sequencing and transmission electron microscopy (TEM). After bed rest, mitochondrial reactive oxygen species (ROS) production increased, whereas mitochondrial respiratory capacity and the abundance of oxidative phosphorylation (OXPHOS) complexes and respiratory supercomplexes’ proteins were preserved. Reduced DRP1 phosphorylation at serine 637 was observed, whereas the abundance of other mitochondrial fusion and fission proteins remained unchanged. TEM analysis revealed preserved mitochondrial morphology, however there was a reduction in mitochondrial volume density indicative of reduced mitochondrial mass. Transcriptomic profiling identified >3000 differentially expressed genes (DEGs), including the downregulation of oxidative phosphorylation‐related transcripts and alterations in mitophagy, antioxidant and oxidoreductase pathways.

## Introduction

Skeletal muscle is a highly dynamic tissue, with its primary functions being the maintenance of posture and movement through contractile activity, and the facilitation of various crucial metabolic processes (Frontera & Ochala, 2015). Skeletal muscle health is a critical determinant of overall well‐being, especially in older adults, where age‐related loss of muscle mass and strength contributes to metabolic and physical impairments. Physical inactivity, either short term or prolonged, experienced due to hospitalisation, chronic illness or injury, can further exacerbate this decline (Coker & Wolfe, 2012; Ikezoe et al., 2012). Moreover periods of inactivity in older adults contribute to diminished functional abilities and mobility, loss of independence and a higher risk of falls and fractures (McKendry et al., 2024; Nunes et al., 2022; Shumway‐Cook et al., 2005; Suetta et al., 2013).

The proper function and health of skeletal muscle are deeply reliant on mitochondria. As the primary site of oxidative phosphorylation‐mediated ATP production within muscle cells, mitochondria support both contractile activity and the metabolic processes essential for muscle maintenance (Gouspillou & Hepple, 2016). The capacity of skeletal muscle to generate and sustain force is highly dependent on mitochondrial function, particularly through oxidative phosphorylation and ATP production (Hargreaves & Spriet, 2020). Although mitochondria are commonly regarded as the ‘powerhouses’ of the cell due to their role in energy production, research over the past two decades has revealed their involvement in a variety of other critical cellular processes. These include autophagy, calcium (Ca^2^^+^) homeostasis, reactive oxygen species (ROS) signalling and the regulation of apoptosis (Anagnostou & Hepple, 2020; Banoth & Cassel, 2018; Protasoni & Zeviani, 2021; Rasola & Bernardi, 2007; Rizzuto et al., 2012). Given these diverse functions of mitochondria in skeletal muscle cell physiology, it is not surprising that their dysfunction has been linked to ageing and inactivity. These include, for example, the age‐related alterations in mitochondrial bioenergetics and susceptibility to permeability transition, changes in quality control and morphology (S Bell & Tower, 2021; Gonzalez‐Freire et al., 2018; Holloway et al., 2018; Joseph et al., 2012; Leduc‐Gaudet et al., 2015; Picard et al., 2010; Rossi et al., 2026; Short et al., 2005), as well as alterations in mitochondrial properties following disuse (Brocca et al., 2012; Eggelbusch et al., 2024; Miotto et al., 2019; Noone et al., 2023; Zuccarelli et al., 2021; Zuccarelli, De Martino et al., 2025). Although the research on disuse and mitochondrial dysfunction has mostly focused on younger, healthy populations, fewer studies have investigated the consequences of physical inactivity on mitochondria specifically within older populations. For instance, 2 weeks of one‐leg immobilisation has been shown to increase mitochondrial H_2_O_2_ emissions (a marker of oxidative stress) in both young and older individuals (Gram et al., 2015). Another study examining the effects of 14 days of 6° head‐down tilt bed rest found that it caused muscle atrophy, decreased mitochondrial content and increased mitochondrial ROS production in older adults (Dulac et al., 2024). Moreover it was shown that elderly individuals experienced a greater reduction in mitochondrial content and biogenesis markers following 2 weeks of bed rest, and their response to rehabilitation was less pronounced compared to younger individuals (Buso et al., 2019). These findings underscore the age‐related differences in mitochondrial dynamics during periods of inactivity and suggest that although recovery is possible, it may be less robust in older populations.

To further explore the effects of inactivity on mitochondrial health in older adults, this study investigated the impact of a 10‐day bed rest period on skeletal muscle mitochondrial function, morphology and gene expression. Although longer‐term disuse models have provided valuable insights, we chose a 10‐day period because it simulates a more clinically relevant scenario, such as recovery from illness or hospitalisation, which typically involves short to medium periods of inactivity. Our primary objective was to examine how this short period of inactivity affects mitochondrial function, ROS production, as well as mitochondrial dynamics and morphology in older adults.

## Methods

### Ethical approval

The study followed the ethical guidelines from the latest update of the Declaration of Helsinki and was approved by the National Ethical Committee of the Slovenian Ministry of Health on 21 July 2023 (reference number 0120–123/2023/9). Before taking part, all participants were given clear information about the study's purpose, procedures and any potential risks, and they provided written consent. After signing the consent form, they could join the study, with the option to withdraw at any time.

### Participants

Ten older men (mean age: 68.5 ± 2.6 years; height: 172.7 ± 6.3 cm; body mass: 85.6 ± 12.3 kg) with no significant comorbidities were involved in the study. Prior to participation, they all underwent medical screening. Exclusion criteria included regular smoking, habitual drug use, blood clotting disorders, a history of deep vein thrombosis with D‐dimer levels over 500 µg L^−1^, as well as acute or chronic skeletal, neuromuscular, cardiovascular or metabolic conditions with complications. Participants were also excluded if they had a previous history of embolism, inflammatory or psychiatric disorders, epilepsy; participated in professional‐level sports; or had ferromagnetic implants.

### Experimental protocol

The study was conducted at Izola General Hospital (Izola, Slovenia), where participants completed 10 days of horizontal bed rest. Baseline measurements (BR0), including muscle biopsies from the *vastus lateralis* and mitochondrial respiration ex vivo, were performed before the bed rest period. Post‐bed rest measurements (BR10) were gathered on the ninth day: muscle biopsies and mitochondrial respiration ex vivo were collected on day nine. During the entire bed rest period, participants remained in a horizontal position, with no physical activities involving muscle contraction allowed. They were provided four meals a day, following an individually tailored eucaloric diet consisting of 60% carbohydrates, 25% fats and 15% proteins, with water provided *ad libitum*. The energy needs of each participant were calculated by multiplying their resting energy expenditure by 1.2 for the bed rest period and 1.4 for the ambulatory period (i.e. the hospital‐based pre‐bed rest period during which the baseline measurements were performed) (Biolo et al., 2008).

### Skeletal muscle tissue collection

Skeletal muscle samples of *musculus vastus lateralis* (approximately 150 mg) were collected through biopsy using a Weil‐Blakesley conchotome (Gebrüder Zepf Medizintechnik GmbH & Co. KG, Dürbheim, Germany) under local anaesthesia (2% lidocaine) before (PRE) and after (BR10) the bed rest intervention: from the right leg at PRE and the left leg at BR10. The biopsies were performed in the morning while participants were fasting, at 9 a.m. for both PRE and BR10. After collection the muscle samples were dissected free of fat and connective tissue under magnification (70×) (OPTIKA − SZM‐LED2, Italy) and subsequently divided into portions. A small portion of approximately 2.0−6.0 mg of the sample was placed into BIOPS solution (in mm: 2.77 CaK_2_ EGTA, 7.23 K_2_ EGTA, 20 imidazole, 20 taurine, 6.65 MgCl_2_, 5.77 ATP, 3.95 phosphocreatine, 0.5 dithiothreitol, 50 K‐Mes, pH 7.1 at 0°C) and immediately used for ex vivo mitochondrial respiration measurements using high‐resolution respirometry (Pesta & Gnaiger, 2012). Two additional 20 mg portions were snap‐frozen and stored at ‐80°C for subsequent use in western blot analysis and blue native gel electrophoresis. Another 5 mg portion was fixed overnight with 2.5% glutaraldehyde + 2% paraformaldehyde in 0.1 m sodium cacodylate buffer and then stored in 0.1 m sodium cacodylate buffer at 4°C until further processing for transmission electron microscopy (TEM).

### High‐resolution respirometry (ex vivo respiration and ROS production)

Measurements were performed in duplicate or quadruplicate. Fibre bundles were separated with sharp‐ended needles under magnification. After this fibre bundles were incubated into 2 mL of BIOPS for 10 min and then in a BIOPS solution containing 50 µg mL^−1 s^aponin for 30 min at 4°C with continuous gentle stirring to ensure complete permeabilization. Samples were washed for 10 min with the respiration medium (MIR05; Oroboros Instruments, Innsbruck, Austria) containing 0.5 mm EGTA, 60 mm potassium lactobionate, 3 mm MgCl_2_ 6H_2_O, 20 mm taurine, 10 mm KH_2_ PO_4_, 20 mm HEPES, 110 mm sucrose and 1 g L^−1^ BSA, pH 7.1, weighed in a balance‐controlled scale (Shimatzdu, Kyoto, Japan). Permeabilized fibres were then measured for wet weight and immediately transferred into the respirometer (Oxygraph‐2k Oroboros Instruments) chambers for O_2_ consumption and hydrogen peroxide (H_2_O_2_) emission analysis. O_2_ consumption and H_2_O_2_ emission were determined in MIR05 supplemented with 20 µm Amplex Red (Invitrogen, Carlsbad, CA, USA), 5 U mL^−1 ^horseradish peroxidase 40 U ml^−1 ^superoxide dismutase (SOD) as previously shown (Dirks et al., 2020; Holloway et al., 2018). Experiments were also performed in presence of myosin II‐ATPase inhibitor (Blebbistatin, 25 µm, dissolved in DMSO 5 mm stock) (Perry et al., 2011) to prevent spontaneous contraction in the respiration medium. Lights in the chambers were kept off during the experiments. Mitochondrial function was evaluated by measuring O_2_ consumption polarographically by high‐resolution respirometry (Pesta & Gnaiger, 2012) and H_2_O_2_ emission fluorometrically by a fluorometry modular attachment (Oroboros Instruments). Data were digitally recorded using DatLab4 software (Oroboros Instruments). Experiments were performed in 2 mL of air‐saturated respiration medium (MIR05) at 37°C. Standardized instrumental and chemical calibrations were performed to correct for back‐diffusion of O_2_ into the chamber from the various components (e.g. leak from the exterior, O_2_ consumption by the chemical medium and by the sensor O_2_) (Pesta & Gnaiger, 2012). Background H_2_O_2_ calibration was performed daily for calculating chemical background fluorescence by titrating various and known H_2_O_2_ concentrations before adding the samples. Afterwards the sensitivity of H_2_O_2_ was checked with multiple H_2_O_2_ titrations at the beginning (with the samples), intermittently and in various respiratory states and at the end of the experiments. The O_2_ concentration in the chamber was maintained between 280 and 400 µm (average O_2_ partial pressure 250 mmHg) to avoid O_2_ limitation of respiration. Intermittent reoxygenation steps were performed during the experiments by injections of pure oxygen. A substrate‐uncoupler‐inhibitor‐titration protocol, with a substrate combination that matches physiological intracellular conditions, was applied (Pesta & Gnaiger, 2012; Zuccarelli et al., 2021). Non‐phosphorylating resting mitochondrial respiration was measured in presence of malate (4 mm) and glutamate (10 mm) (‘leak’ respiration). Succinate (10 mm) was added to support convergent electron flow into the Q‐junction through complexes I and II and to maximized H_2_O_2_ emission (Holloway et al., 2018). This was followed by submaximal titration of ADP (12.5, 25, 175, 250, 500, 1000, 2000, 4000, 6000, 8000, 10,000 µm) to assess complex I+II‐linked ADP sensitivity and maximal ADP‐stimulated mitochondrial respiration (OXPHOS). Maximal uncoupled mitochondrial respiration (electron transport system capacity, ETS) was measured by stepwise additions of chemical uncoupler protonophore carbonylcyanide‐*p* trifluoromethoxyphenylhydrazone (FCCP). Rotenone (1 µm) was added to inhibit complex I. Cytochrome C (10 µm) was added to test mitochondrial outer membrane integrity. The addition of cytochrome C had no significant additive effects on respiration, with minor increases of <5%, thereby confirming the integrity of the outer mitochondrial membrane. Finally antimycin A (2.5 µm) was added to inhibit complex III, providing a measure of residual O_2_ consumption, indicative of non‐mitochondrial O_2_ consumption. Mitochondrial respiration indices were then corrected for O_2_ flux resulting from residual O_2_ consumption. The degree of coupling of oxidative phosphorylation for a specific substrate supply (glutamate, malate and succinate) was determined by calculating the ratio between OXPHOS and LEAK respiration (Pesta & Gnaiger, 2012). ADP sensitivity was analysed according to a two‐sequential hyperbolic function approach (Zuccarelli, De Martino et al., 2025), allowing the characterization of distinct phases of ADP‐dependent respiratory kinetics. Apparent Km was calculated as the ADP concentration corresponding to 50% of maximal respiration. The obtained mitochondrial respiration values were also normalized by citrate synthase (CS) activity (see below), taken as an estimated index of mitochondrial content (Larsen et al., 2012).

### CS activity analysis

Muscle samples used for high‐resolution respirometry analysis were homogenized in CelLyticTM MT Cell lysis reagent (C3228 Merck). The lysates were kept on ice for 30 min and then centrifuged at 14,000 *g* for 10 min at 4°C. The supernatant was collected for protein quantification according to the method of Lowry et al. (1951). Protein extracts (5, 10, 15 µg) were added to each well of a 96‐well‐microplate containing 100 µL of 200 mm Tris, 20 µL of 1 mm 5, 5’‐dithiobis‐2‐nitrobenzoate (DTNB), freshly prepared 6 µL of 10 mm acetyl‐coenzyme A (Acetyl‐Co‐A) and water to a final volume of 190 µL. To assess endogenous acetylase activity, background ΔAbs was recorded for 90 s at 10 s intervals at 412 nm and 25°C using a CLARIOstar microplate reader (BMG LABTECH). The reaction was initiated by adding 10 µL of 10 mm oxaloacetic acid (OAA), and absorbance was monitored for an additional 90 s at 10 s intervals. Background ΔAbs values were subtracted from those obtained after OAA addition. All assays were performed in triplicate at 25°C. Enzyme activity was expressed as µmol min^−1^ (mU).

### Skeletal muscle lysate preparation and western blotting

Frozen tissue samples were placed in Pierce^TM^ RIPA buffer (#89901, ThermoFisher Scientific, Waltham, MA, USA) containing Halt^TM^ protease and phosphatase inhibitor cocktail (#1861281, ThermoFisher Scientific). Samples were homogenized using the Velp Scientifica LS Overhead Stirrer (Velp Scientifica, Usmate, Italy) at speed 5 for 30 s thrice, and then left on ice for 10 min. Then samples were centrifuged at 15,200 *g* for 10 min at 4°C (Frontier™ 5000 Micro Series centrifuge, FC5515R, OHAUS, Parsippany, NJ, USA). The Pierce BCA Protein Assay Kit (#23225, ThermoFisher Scientific) was used to assess total protein concentration in collected supernatants with a microplate reader (Tecan Infinite 200 Pro, Tecan, Männedorf, Switzerland) at 560 nm. Samples mixed with Laemmli buffer were loaded onto a precast 3%–8% Tris‐Acetate polyacrylamide gels (#EA03785BOX, ThermoFisher Scientific), or 4%−12% Bis‐Tris polyacrylamide gels (#NP0336BOX, ThermoFisher Scientific) or home‐made 15% SDS‐PAGE gels (to separate the two forms of Cyclophilin D). PageRuler™ Plus Prestained Protein Ladder (#26619, ThermoFisher Scientific) was used as a molecular weight marker. Gel‐electrophoresis was performed at 4°C in an XCell *SureLock*™ Mini‐Cell mini‐protein gel electrophoresis system (#EI0001, Invitrogen, Waltham, MA, USA) at 60 mA (30 mA per gel) until the run was complete. Subsequently proteins were transferred to a nitrocellulose membrane at 100 V for 90 min at 4°C using the Mini Trans‐Blot cell (#1703930, Bio‐Rad, Hercules, California, USA). The membrane was then blocked with a 5% blocking solution (either nonfat dry milk or bovine serum albumin) for 60 min at room temperature. After blocking the membrane was washed with phosphate‐buffered saline containing 0.05% Tween (PBS‐T) and incubated with primary antibodies overnight at 4°C. The next day the membrane was washed in PBS‐T (3 × 5 min) and incubated with secondary antibodies for 60 min at room temperature. The following primary antibodies have been used: Mouse total OXPHOS Human WB Antibody Cocktail (NDUFB8, COX II, SDHB, UQCRC2, ATP5A) (Abcam, Cambridge, UK; #ab110411) at a dilution of 1:1000; rabbit optic atrophy 1 (OPA1) (Abcam; #ab42364) at 1:700; mouse dynamin‐like protein 1 (DRP1) (BD Biosciences, Franklin Lakes, NJ, USA; #611113) at 1:1000; rabbit phospho‐DRP1 (DRP1 S637) (Cell Signalling Technology, Danvers, MA, USA; #4867) at 1:500; mouse mitofusin 2 and mitofusin 1 antibody cocktail (MFN2, MFN1) (Abcam; #ab57602) at 1:1000; Rabbit PTEN‐induced kinase 1 (PINK1) (Cell Signalling Technology; #6946) at 1:1000; rabbit parkin (Cell Signalling Technology; #2132) at 1:1000; rabbit β‐actin (Abcam; #ab8227) at 1:1000; mouse cyclophilin F (CyPD) (Abcam; #ab110324) at 1:2000; mouse anti‐ATP5A antibody (Abcam; #ab14748) at 1:500; and rabbit FIS1 (Proteintech, Rosemont, IL, USA; #10956‐1‐AP) at 1:2000. For the analysis of OXPHOS proteins, samples were heated to 37°C prior to loading to preserve antibody binding, as recommended for the OXPHOS antibody cocktail. Western blotting for OXPHOS proteins was performed first. Subsequently samples were boiled at 95°C for 5 min, and western blot analysis of all other proteins was carried out. Secondary antibodies were anti‐mouse 1:2000 (#GTX213111‐01, GeneTex, Irvine, CA, USA), or anti‐rabbit 1:2000 (#GTX21311001, GeneTex). The membrane was then washed in PBS‐T again (3 × 5 min). Immobilon Crescendo Western HRP Substrate (#WBLUR0500, Millipore, Burlington, Massachusetts, USA) was used for detecting protein bands. iBright FL1500 Imaging System (#A44241, ThermoFisher Scientific) recorded the chemiluminescent signal, and ImageJ software was used to analyse the ratio of each protein staining intensity to the staining intensity of β‐actin that was used as a loading control. Alternatively anti‐mouse fluorescent secondary antibodies were used (IRDye 680 and IRDye 800) (LI‐COR Biosciences, Lincoln, NE, USA) diluted 1:10,000 and incubated for 2 h; afterward the signals were detected with an Odyssey CLx scanner (LI‐COR Biosciences) and quantified using ImageStudio software (LI‐COR Biosciences).

### Isolation of mitochondria from skeletal muscle and blue‐native polyacrylamide gel electrophoresis

Mitochondrial proteins were extracted from 20 mg of muscle tissue following previously published protocols (Fernandez‐Vizarra & Zeviani, 2021; Frezza et al., 2007) by digitonin permeabilization and differential centrifugation. Briefly samples were homogenized with a Velp Scientifica LS Overhead Stirrer at speed 5 for 15 s (twice) in isolation buffer 1 (6.7 mL of 1 m sucrose, 5 mL of 1 m Tris‐HCl, 5 mL of 1 m KCl, 1 mL of 1 m EDTA, 2 mL of 10% BSA; pH adjusted to 7.4, volume brought to 100 mL with distilled water). Homogenates were centrifuged (Frontier™ 5000 Micro Series centrifuge, FC5515R, OHAUS) at 700 g for 10 min at 4°C. The supernatant was then centrifuged at 10,000 *g* (Frontier™ 5000 Micro Series centrifuge, FC5515R, OHAUS) for 10 min, and the pellet was resuspended in buffer 2 (25 mL of 1 m sucrose, 3 mL of 0.1 m EGTA/Tris, 1 mL of 1 m Tris‐HCl; pH 7.4, volume adjusted to 100 mL with water). Protein concentration was measured by Bradford assay (#23238, Pierce™ Bradford Plus Protein Assay Reagent, ThermoFisher Scientific).

For blue‐native polyacrylamide gel electrophoresis (BN‐PAGE) analysis, 30 µg of mitochondrial protein was mixed with digitonin (4 mg per milligram of protein), NativePAGE Sample Buffer (#BN2003, ThermoFisher Scientific) and incubated for 10 min, followed by centrifugation at 15,200 *g* for 30 min. Supernatants were mixed with Coomassie to its final concentration of 0.18% in the sample and loaded onto NativePAGE gels (#BN1001BOX, ThermoFisher Scientific), run using commercially available buffers (#BN2007, NativePAGE™ Running Buffer Kit ThermoFisher Scientific) at 100 V for 1 h and then at 250 V for 2.5 h at 4°C, and subsequently blotted as described in the previous section. NativeMark Unstained Protein Standard (#57030, ThermoFisher Scientific) was used as a molecular weight marker.

Mitochondrial supercomplexes containing subunits of complex I were detected using a rabbit antibody against complex I protein NDUFS1 1:1000 (#Ab169540, Abcam), followed by incubation with a secondary antibody 1:2000 (#GTX213110‐01, GeneTex). Complex I protein was selected for detection because it is a central component of all major mitochondrial supercomplexes, making it a reliable marker for their presence (Jha et al., 2016). Subsequently SDHA (complex II) was detected using a mouse antibody against SDHA 1:1000 (#Ab14715, Abcam), followed by incubation with a secondary antibody (1:2000; #GTX213111‐01, GeneTex), and used as loading control.

### TEM sample preparation, imaging and analysis

The processing and imaging of the samples were carried out by the Imaging Facility of the Department of Biology, University of Padua (Padua, Italy). As previously mentioned the samples were fixed overnight at 4°C in 2.5% glutaraldehyde and 2% paraformaldehyde in 0.1 m sodium cacodylate buffer (pH 7.4) and subsequently stored in 0.1 m sodium cacodylate buffer at 4°C until postfixation. The samples were post‐fixed in 1% osmium tetroxide in 0.1 M sodium cacodylate buffer for 1 h at 4°C. Afterward they were dehydrated through sequential 10‐min incubations in 10%, 25%, 50% 75%, and 95% ethanol solutions, followed by three 10‐min incubations in 100% ethanol. Next the samples underwent three 15‐min incubations with propylene oxide, followed by a 2‐h incubation with a 1:1 mixture of propylene oxide and EMbed 812 (Electron Microscopy Sciences, Hatfield, PA, USA) at 37°C. Finally the samples were embedded in EMbed 812 (Electron Microscopy Sciences) epoxy resin. Ultrathin sections were obtained with a Leica EM UC7 (Leica Microsystems, Wetzlar, Germany) ultramicrotome and subsequently counterstained with uranyl acetate and lead citrate. Samples were then examined with a Tecnai G2 transmission electron microscope (FEI‐Thermo Fisher, Waltham, MA, USA) operating at 120 kV, digital images were acquired using a Veleta digital camera (Olympus Soft Imaging Solutions, Münster, Germany). Longitudinally oriented fibres were photographed at ×13,000 magnification (images 2048*2028 pixels, where the length of each pixel is 3.6 nm), with at least 15 images taken from the subsarcolemmal (SS) region (total of 135 images) and at least 15 images from the intermyofibrillar region (IMF) (total of 125 images).

#### Mitochondrial volume density

The mitochondrial volume density was assessed by point counting (Gamboa & Andrade, 2010; Motanova et al., 2026) using a grid size of 150 nm (2500 vertices grid). Intermyofibrillar mitochondria were expressed per myofibrillar space (µm^3^ µm^−3^) using the following formula Mito_VD IMF = _(vertices that touch mitochondria)/(total myofibrillar space vertices), and subsarcolemmal (SS) mitochondrial content was expressed relative to the surface area of the outermost myofibril (µm^3^ µm^−2^) using the following formula Mito_VD SS_ = (vertices × 0.01 µm^2^)/(length of myofibrils in µm). The average volume fraction of mitochondria was then calculated using the following formula: Total Mito_VD_ = Mito _VD IMF_ + (Mito_VD SS_/20).

#### Mitochondrial morphology

Mitochondria were manually traced, and predefined shape descriptors were obtained using ImageJ (1.52v; National Institutes of Health, Bethesda, MD). Mitochondrial morphological variables were studied from two different mitochondrial fractions (SS and IMF). Based on the previously published approach we measured aspect ratio (AR) and form factor (FF) (Motanova et al., 2026). The AR, which is the ratio of a mitochondrion's major to minor axis, provides a measure of its elongation. In contrast the FF, calculated as (perimeter^2^)/(4π·surface area), captures morphological complexity and the extent of mitochondrial branching (Picard et al., 2013).

Two independent, blinded operators conducted all mitochondrial analyses, including mitochondrial volume density and morphology, without knowledge of participant ID or timepoint. To evaluate operator‐related variability and minimise the risk of bias, we calculated the inter‐operator coefficient of variation (CV). The inter‐operator CVs were 3.6% for AR, 2.0% for FF and 5.9% for mitochondrial VD. During mitochondrial morphology analyses all mitochondria within the selected images were quantified. The total number of mitochondria identified by the two operators showed an inter‐operator CV of 5.7%.

### RNA extraction, RNA sequencing and bioinformatic analysis

Frozen muscle samples (15 mg) were placed into 2 mL tubes, along with 500 µL of TRIzol™ Reagent (#15596018, Invitrogen, Waltham, MA, USA) and stainless‐steel beads (5 mm mean diameter, #69989, Qiagen, Hilden, Germany). The samples were then lysed using a Tissue Lyser II (Qiagen) for 2.5 min at 30 Hz. Following lysis, 100 µL of chloroform was added, and the samples were briefly vortexed. They were then incubated on ice for 15 min before being centrifuged at +4°C for 10 min at 14,000 *g*. The aqueous phase (top fraction) was carefully collected and purified using the Norgen Total RNA Purification Kit (#37500, Norgen Biotek, Thorold, Canada).

RNA concentration was measured using a Qubit fluorometer (Thermo Fisher Scientific), whereas RNA integrity was assessed using TapeStation (Santa Clara, CA, USA), an automated electrophoresis system. All samples exhibited an RNA integrity number (RIN) greater than 7. The libraries were sequenced using a paired‐end (PE) method (150 nucleotides per read) on a NextSeq 550 (Illumina, San Diego, CA, USA), achieving a median sequencing depth of 20 million reads per sample.

Raw sequencing data were processed using the DESeq2 (version 1.42.0) pipeline in R (version 4.3.2). Only the coding‐sequence (CDS) count matrix was used for differential expression analysis. Genes with fewer than 10 total counts across all samples were removed prior to modelling. Count normalisation and dispersion estimation were performed using DESeq2's internal methods. To account for the paired structure of PRE and BR10 samples within each participant, differential expression analysis was performed using a design formula including participant identity and time point (design = ∼ Subject + TimePoint), where TimePoint had two levels (PRE and BR10). *P*‐values were adjusted using the Benjamini‐Hochberg method, and genes with padj < 0.05 were considered significantly differentially expressed. No log_2_FC threshold was applied in the statistical model. Log_2_ fold‐change thresholds were applied only for descriptive classification of effect size and for visualisation in the volcano plot.

Variance‐stabilising transformation (VST) or regularised log transformation (rlog) was applied to normalised counts depending on the downstream analysis (VST: Principal Component Analysis (PCA) and gene‐of‐interest plots; rlog: heatmaps). Quality control included the inspection of sample clustering by PCA and assessment of sample‐to‐sample distances, which did not reveal batch effects or outliers. Gene Ontology (GO) enrichment analysis was performed on significant DEGs using the clusterProfiler package and the org.Hs.eg.db annotation database. Biological Process (BP) and Cellular Component (CC) ontologies were tested with *q* < 0.05 considered significant. For visualisation the top 10 enriched terms were shown. To assess enrichment in pathways relevant to mitochondrial function and oxidative stress, DEGs were tested against curated gene sets (mitochondrial biogenesis, mitophagy, oxidoreductase activity and antioxidant activity). Fisher's exact test was used for overrepresentation analysis with the background set defined as all expressed CDS genes. *P*‐values were adjusted using the Benjamini‐Hochberg method, and odds ratios were reported. Expression patterns of selected genes contributing to these enrichments were visualised as pathway‐focused log_2_ fold‐change barplots, in which blue bars indicate genes with padj < 0.05 and grey bars indicate genes that did not reach statistical significance individually but contributed to pathway‐level enrichment.

### Statistical analysis

Results are expressed as means ±SD. The Shapiro‐Wilk test and visual inspection of Q‐Q plots were used to assess the normality of the data. Parameters that were not normally distributed were analysed using the non‐parametric Wilcoxon test. To account for multiple comparisons within the mitochondrial fission and fusion protein panel, statistical significance was evaluated using a Bonferroni‐corrected significance threshold. Differences were considered statistically significant when *P* ≤ 0.0056. For exploratory correlation analysis of mitochondrial volume density and CS activity, the relationship between variables was evaluated using Pearson correlation and simple linear regression via the least‐squares method, with statistical significance set at *P* ≤ 0.05. Effect sizes were calculated using Cohen's *d* and interpreted using standard thresholds (0.20 = small, 0.50 = medium and 0.80 = large). Statistical analyses were conducted using GraphPad Prism (version 8.0.1 for Windows, GraphPad Software, Boston, MA, USA) and Excel (Microsoft Excel for Microsoft 365 MSO; Version 2412 Build 16.0.18324.20092). All graphs were generated using GraphPad Prism.

## Results

All participants completed the study and all the experimental procedures. There were no reported side effects associated with the biopsy, or bed rest exposure.

Preliminary data from this cohort (Scarpelli et al., 2025) demonstrated that the 10‐day bed rest exposure successfully induced muscle atrophy and functional impairment. Briefly 10 days of inactivity induced a 6.2% reduction in *quadriceps femoris* volume measured by MRI (*P* < 0.001), a 7.4% decrease in *vastus lateralis* muscle thickness assessed by ultrasound (*P* = 0.02), and a 13.3% decrease in strength (*P* < 0.001).

### Mitochondrial respiration

To determine whether short‐term bed rest altered mitochondrial content and respiratory function, CS activity and ex vivo mitochondrial respiration were assessed in permeabilised skeletal muscle fibres.

**Figure 1:**
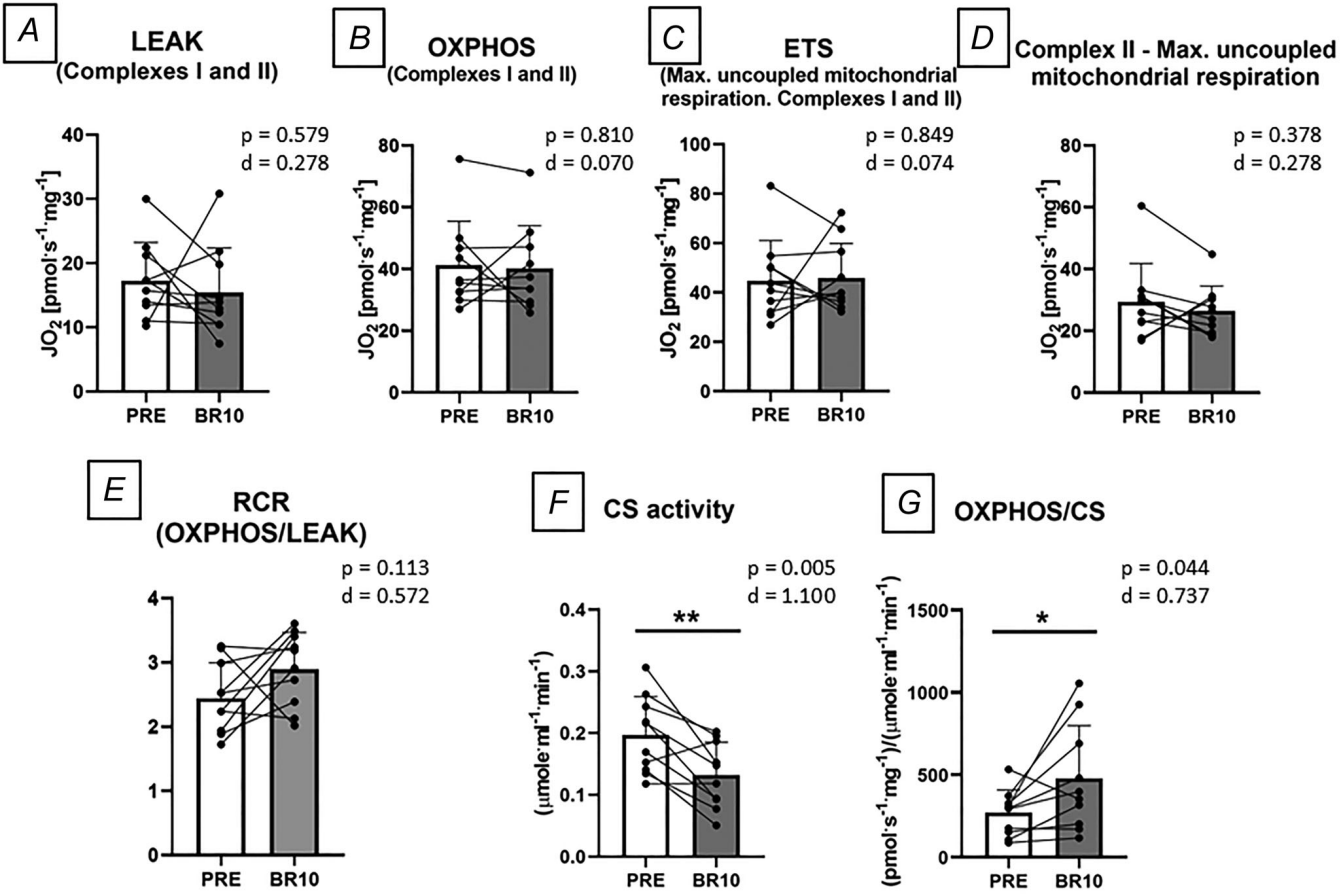
Assessments of ex vivo mitochondrial function and mass Individual and mean (± standard deviation) values of mitochondrial respiration variables obtained by high‐resolution respirometry in permeabilised skeletal muscle fibres are shown; data were obtained before bed rest (PRE) and after 10 days of bed rest (BR10). *A‐D*, LEAK respiration, oxidative phosphorylation (OXPHOS) capacity, electron transport system (ETS) capacity and complex II maximal uncoupled respiration. *E*, respiratory control ratio (OXPHOS/LEAK) data; *F*, citrate synthase (CS) activity; and *G*, OXPHOS/LEAK. All data were analysed using the two‐tailed paired *t* test, with a sample size of *n* = 10. Effect sizes were calculated using Cohen's *d*.

Individual and mean (± SD) data of CS activity are shown in Fig. 1. Although CS activity was significantly reduced after bed rest, the main data related to mitochondrial respiration (JO_2_) obtained by high‐resolution respirometry did not change following bed rest. Mitochondrial leak respiration (‘LEAK’), maximal ADP‐stimulated mitochondrial respiration (‘OXPHOS’), supported by complex I and complex II, maximal uncoupled mitochondrial respiration supported by complex I and complex II (‘ETS’) and rotenone‐insensitive ETS, the mitochondrial electron transport system capacity supported by complex II, remain unchanged. Mitochondrial coupling of oxidative phosphorylation (‘respiratory control ratio’), calculated as OXPHOS/LEAK, was not affected by bed rest (Fig. 1).

The obtained mitochondrial respiration values were also normalised by CS activity (Fig. 1*G*), a commonly used biochemical marker of mitochondrial content. OXPHOS/CS ratio increased after bed rest, suggesting an intrinsic improvement in respiratory function.

**Figure 2:**
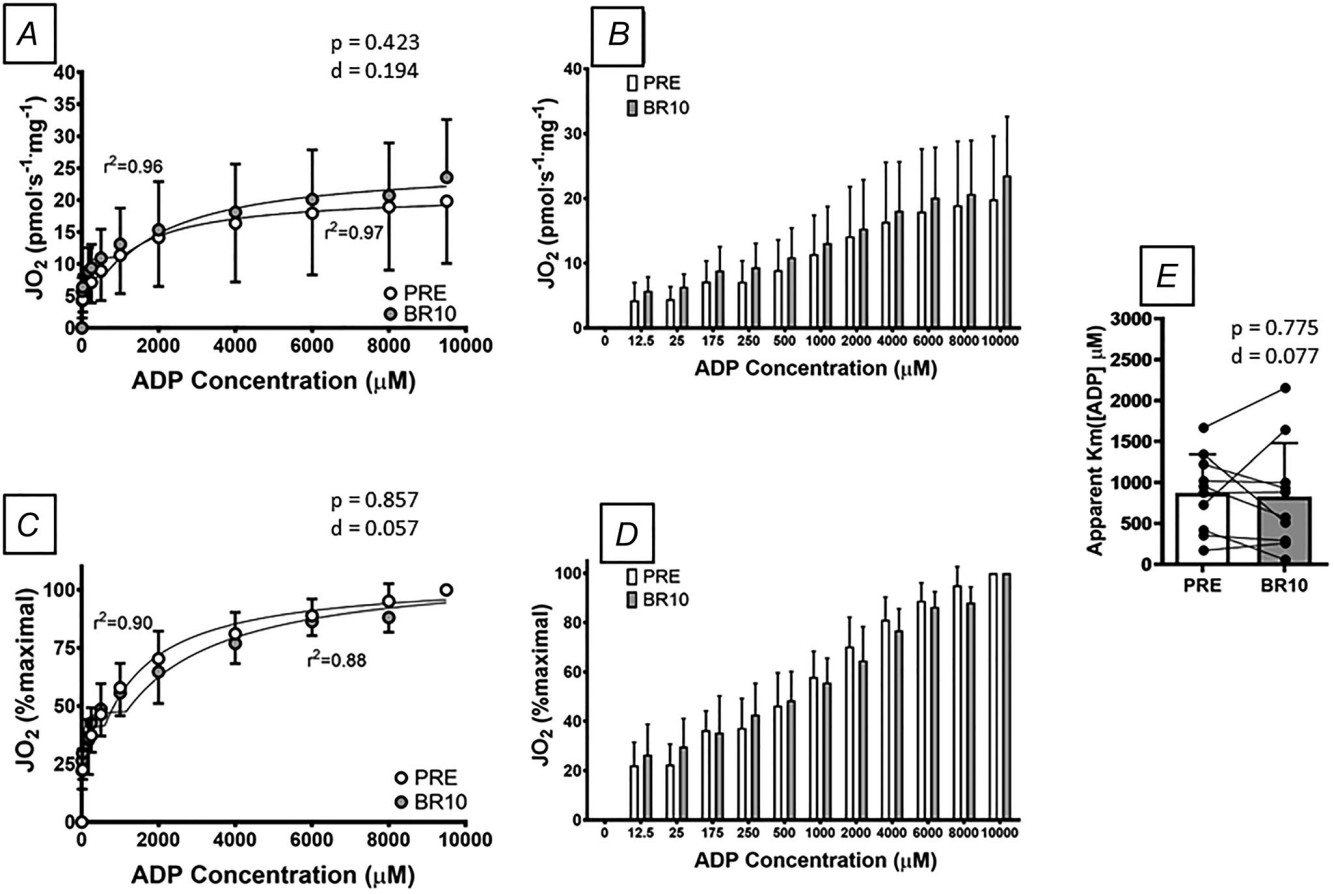
ADP‐stimulated mitochondrial respiration *A,B*, averaged respiration rate (mitochondrial oxygen consumption rate, JO_2_, in absolute terms) data as a function of adenosine diphosphate ([ADP]) before bed rest (PRE) and after 10 days of bed rest (BR10). *C,D*, mitochondrial respiration values expressed as a percentage of maximal values. *E*, the apparent Michaelis constant (*K*_m_), which indicates the ADP concentration ([ADP]) at 50% of maximal mitochondrial oxygen consumption rate (JO_2_max). Data fittings were performed by the least‐squared residuals method, differences among ADP concentrations were assessed using two‐way ANOVA, and apparent *K*_m_ data were analysed using the two‐tailed paired *t* test. Effect sizes were calculated using Cohen's *d*. Sample size: *n* = 10.

We next examined the sensitivity of mitochondrial respiration across a range of submaximal ADP concentrations, including some indicative of resting skeletal muscle (Howlett et al., 1998; Phillips et al., 1996) (Fig. 2). The relative mitochondrial respiration (expressed both in absolute and relative values) in presence of various submaximal ADP concentrations was unchanged following bed rest. Data were analysed according to a two‐sequential hyperbolic function (Zuccarelli, De Martino et al., 2025). The [ADP] values corresponding to 50% of maximal JO_2_, indices of mitochondrial sensitivity, were not affected by bed rest, suggesting that the major control point in the metabolic homeostasis was preserved after 10 days of bed rest and in presence of a reduced mitochondrial content.

### Mitochondrial ROS emission

**Figure 3:**
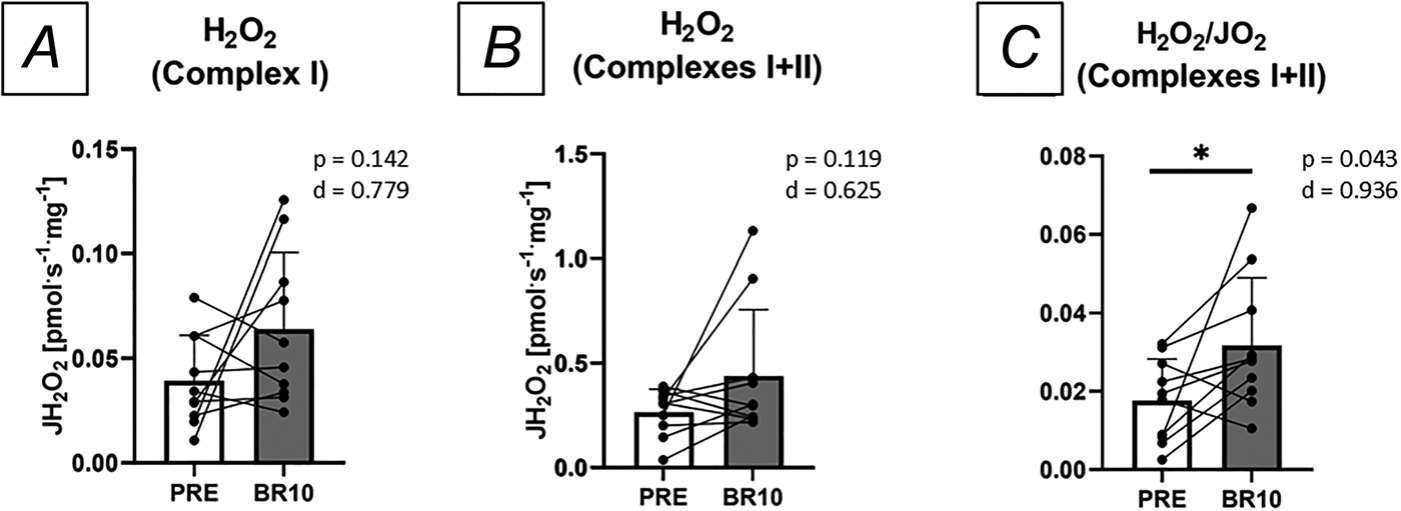
Mitochondrial reactive oxygen species (ROS) emission *A*, maximal hydrogen peroxide (H_2_O_2_) emission linked to complex I; *B*, the same variable linked to complex II. *C*, the ratio between hydrogen peroxide (H_2_O_2_) emission and mitochondrial oxygen consumption rate (JO_2_) in absence of adenosine diphosphate (ADP). All data were analysed using the two‐tailed paired *t* test, with a sample size of *n* = 10. Effect sizes were calculated using Cohen's *d*.

Having observed that mitochondrial respiration was preserved despite reduced mitochondrial mass, we next assessed mitochondrial ROS production. Individual and mean (± SD) data of H_2_O_2_ linked to complex I and complex II before and after bed rest are shown in Fig. 3. H_2_O_2_ was maximised during leak respiration. Maximal H_2_O_2_ emission tended to increase following 10 days of bed rest when both complex I (+62%; *P* = 0.142) and complex II (+65%; *P* = 0.119) were stimulated. The ratio of H_2_O_2_ emission to JO_2_ respiration was increased after bed rest, suggesting an increased maximal ROS production after bed rest. We next examined the H_2_O_2_ emission in response to submaximal (and more physiological) concentrations of ADP.

**Figure 4:**
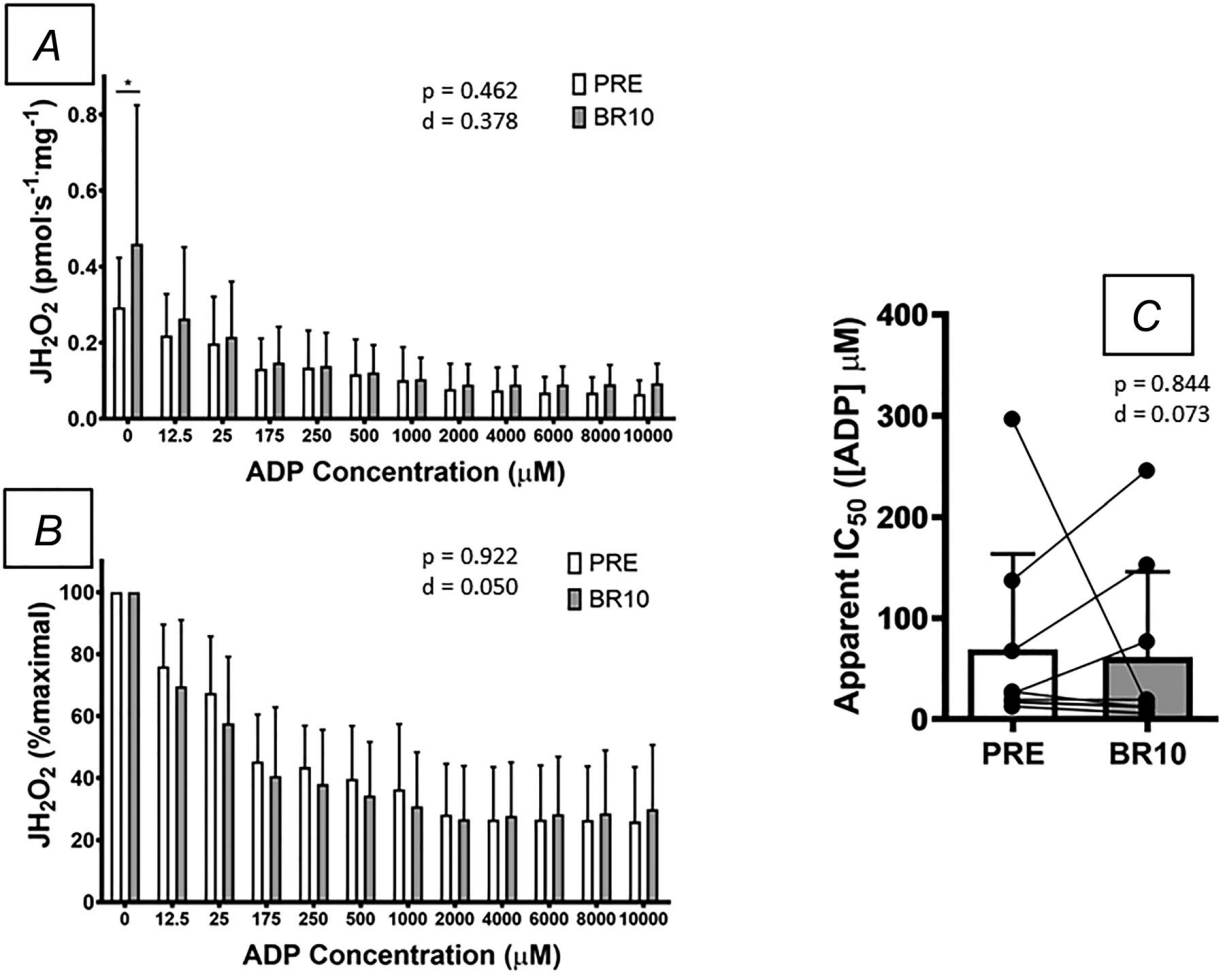
Mitochondrial reactive oxygen species (ROS) emissions in the presence of adenosine diphosphate (ADP) Absolute *A*, and relative *B*, hydrogen peroxide (H_2_O_2_) inhibition following ADP titrations. *C*, the ADP concentration required to decrease H_2_O_2_ emission by 50%. Differences among ADP concentrations were assessed using two‐way ANOVA, and apparent half‐maximal inhibitory concentration (IC_50_) data were analysed using the two‐tailed paired *t* test. Effect sizes were calculated using Cohen's *d*. Sample size of *n* = 10.

In Fig. 4 mitochondrial H_2_O_2_ emission decreases in response to increasing concentrations of ADP in absolute and relative (as 100% of the maximal H_2_O_2_ emission capacity) values before and after bed rest are shown. None of the data increased following bed rest, suggesting an unchanged H_2_O_2_ emission in presence of ADP. Additionally the IC_50_, which indicates the ADP concentration required to reduce H_2_O_2_ emissions by 50%, did not increase after bed rest, further highlighting the unchanged ROS production in presence of ADP in older adults following 10 days of bed rest.

### Abundance of OXPHOS complexes and respiratory supercomplexes

**Figure 5:**
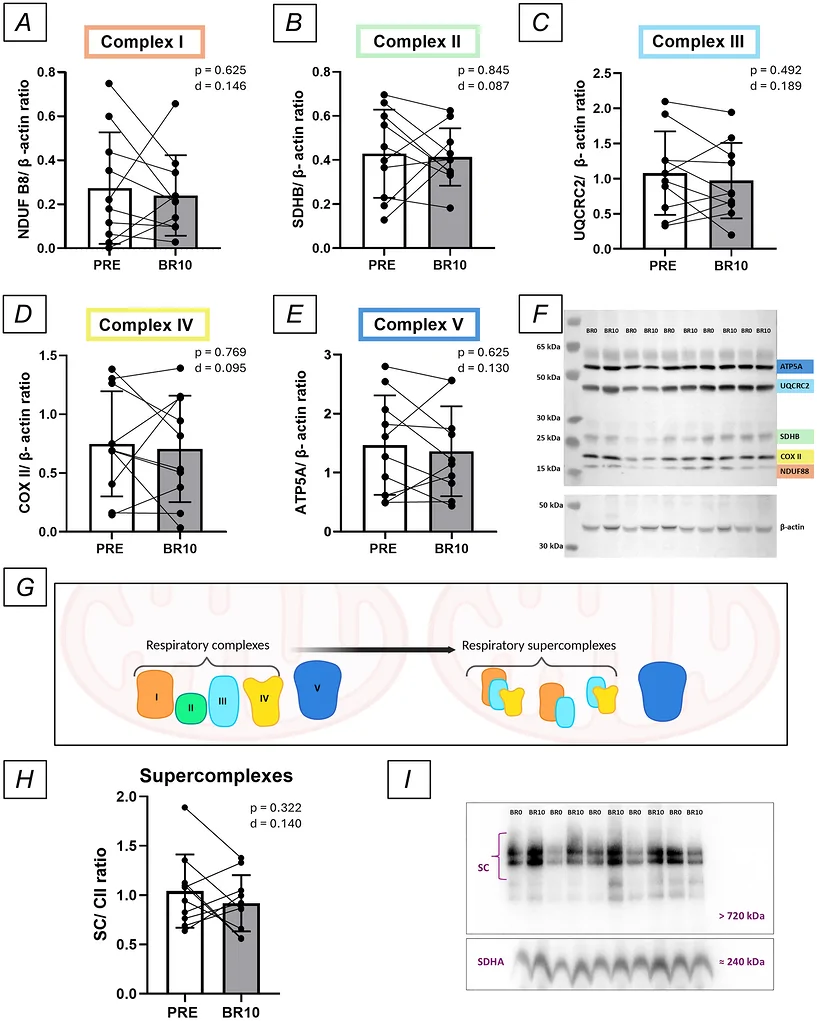
Abundance of mitochondrial oxidative phosphorylation (OXPHOS) complexes and respiratory supercomplexes analysed before bed rest (PRE) and after 10 days of bed rest (BR10) *A‐E*−, graphs of the expression levels of mitochondrial OXPHOS complexes I to V. *F*, a representative western blot of the OXPHOS complexes, with beta‐actin (β‐actin) used as a reference for normalization. *G*, a schematic of how mitochondrial respiratory complexes assemble into supercomplexes; created in BioRender. *H*, a graph depicting the abundance of mitochondrial supercomplexes; *I*, two representative membranes after blue native polyacrylamide gel electrophoresis (BN‐PAGE), displaying the supercomplexes and succinate dehydrogenase complex flavoprotein subunit A (SDHA), to which they were normalized. All data were analysed using the Wilcoxon signed‐rank test, with a sample size of *n* = 10 for *A‐E*, and *n* = 9 for *H*. Effect sizes were calculated using Cohen's *d*.

Following the observation of preserved mitochondrial respiration but increased ROS production, we sought to investigate potential underlying mechanisms by evaluating the abundance of mitochondrial OXPHOS complexes and respiratory supercomplexes. However, no significant differences were found in any of the parameters assessed (Fig. 5).

**Figure 6:**
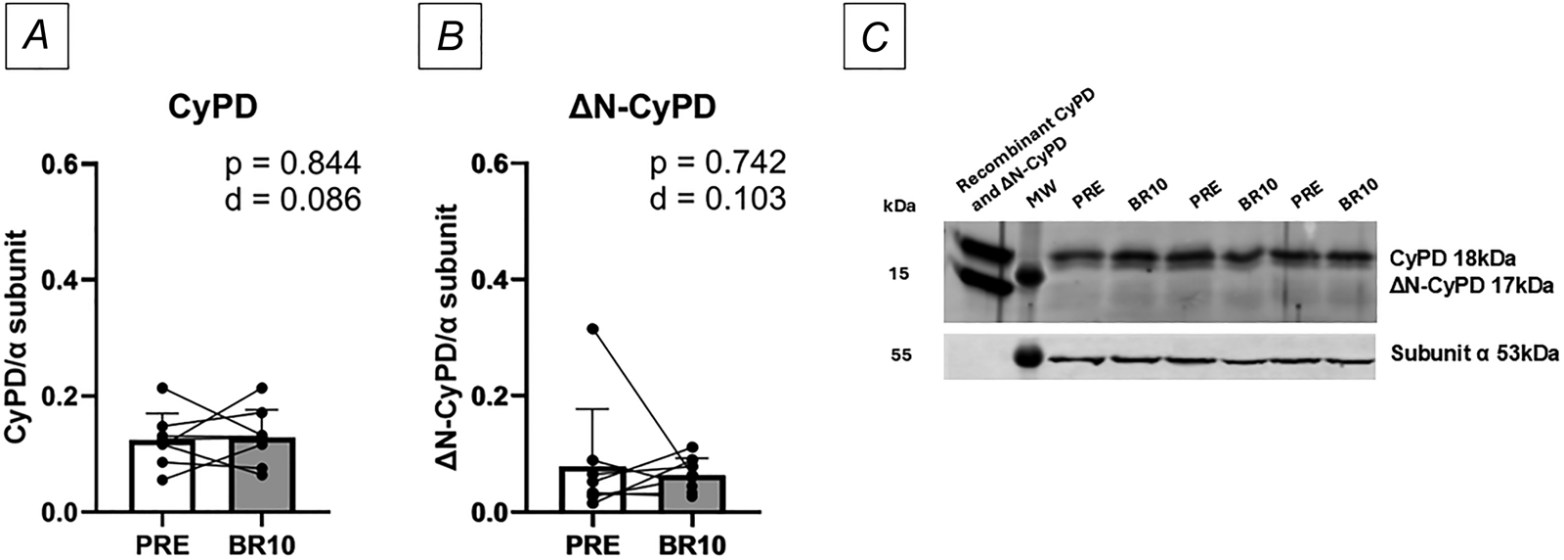
Expression of proteins related to permeability transition in the inner mitochondrial membrane before bed rest (PRE) and after 10 days of bed rest (BR10) *A*, the expression levels of cyclophilin D (CyPD); *B*, the expression of N‐terminally truncated cyclophilin D (ΔN‐CyPD), both relative to the expression levels of the α subunit of mitochondrial ATP synthase (complex V). *C*, representative western blots of all proteins analysed. Molecular weight (MW) is indicated. Recombinant CyPD and ΔN‐CyPD were produced and purified as described in Coluccino et al. (2024). All data were analysed using the Wilcoxon signed‐rank test, with a sample size of *n* = 8. One participant identified as a statistical outlier by Grubbs' test was excluded from the analysis. One participant identified as a statistical outlier by Grubbs' test was excluded from the analysis. Effect sizes were calculated using Cohen's *d*.

To further investigate potential mechanisms underlying the observed increase in ROS production, we assessed mitochondrial functional integrity by examining a key sensitiser of the mitochondrial permeability transition (PT), which is mediated by opening of a Ca^2+^‐dependent non‐selective channel located in the inner mitochondrial membrane, known as the permeability transition pore (PTP), that causes the collapse of the inner membrane potential and rupture of the outer membrane leading to apoptotic and necrotic cell death. Increased ROS levels trigger PTP opening, which further amplifies ROS production through a feedback mechanism known as 580 ROS‐induced ROS release (Bernardi et al., 2023; Rasola et al., 2010). Specifically we analysed the expression of the mitochondrial matrix protein cyclophilin D (CyPD), examining both the full‐length form and a novel form of CyPD lacking the first 13 N‐terminal residues (ΔN‐CyPD), which, according to Coluccino et al. (2024), represents a more active form of the molecule. Both protein levels were related to the complex V content, which remained unchanged after bed rest (Fig. 5*E*). As shown in Fig. 6*A,B*, neither of the CyPD forms changed after bed rest.

### Mitochondrial dynamics

Given the lack of changes in mitochondrial OXPHOS complexes and supercomplexes, we next investigated other potential mechanisms associated with the increased ROS production. We focused on mitochondrial dynamics, specifically proteins involved in fusion, fission and mitophagy. We assessed the expression of key proteins related to mitochondrial fusion (MFN1, OPA1), mitochondrial fission (DRP1 and Fis1), inhibition of mitochondrial fission (DRP1 S637) and mitophagy (PINK1 and Parkin). Of note OPA1 exists in long (L‐OPA1) and short (S‐OPA1) isoforms generated by proteolytic processing. The L‐OPA1/S‐OPA1 ratio reflects the balance between OPA1 isoforms involved in mitochondrial inner membrane remodelling and fusion regulation (Ge et al., 2020; Wang et al., 2021).

**Figure 7:**
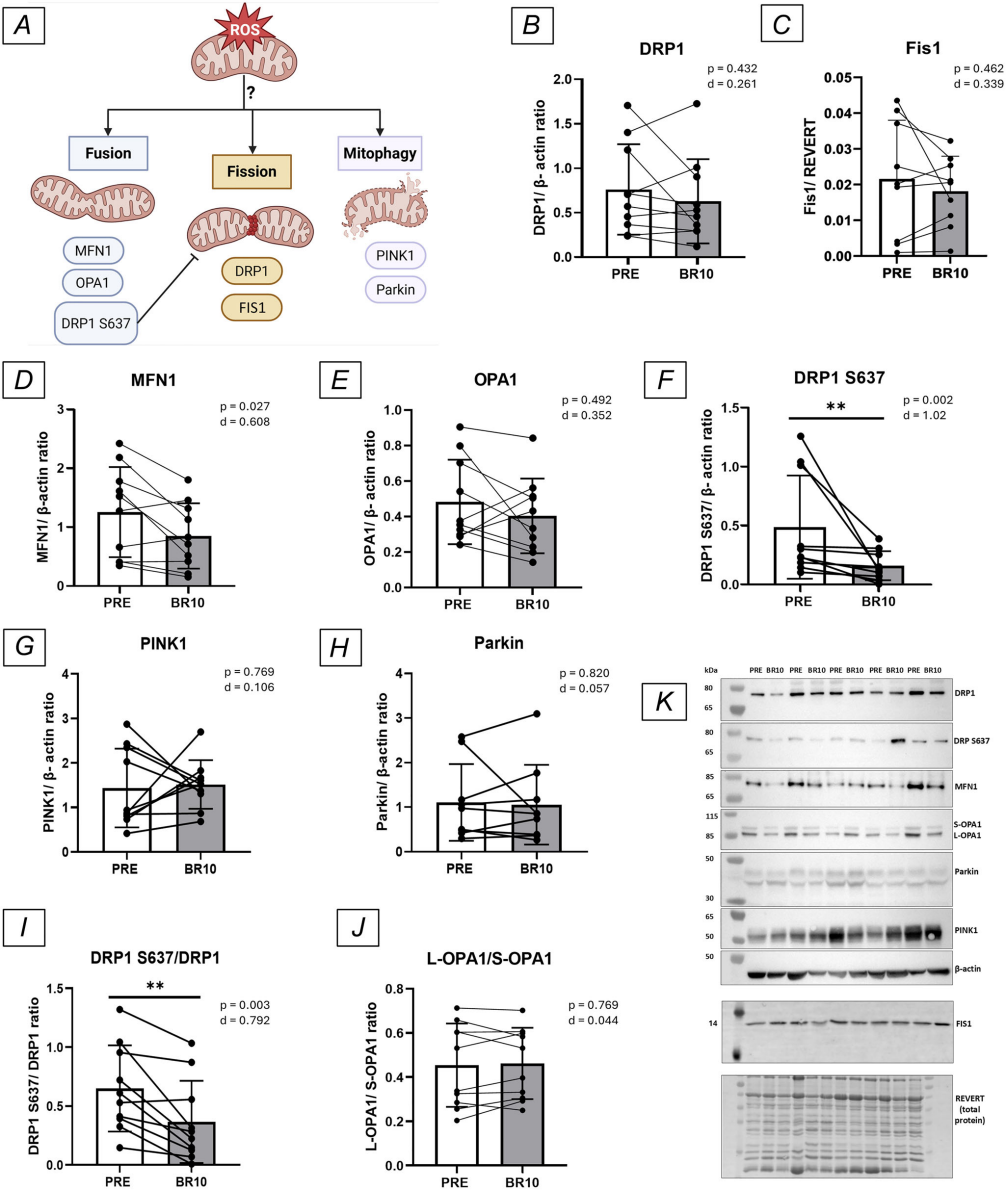
Expression of proteins related to mitochondrial dynamics assessed before bed rest (PRE) and after 10 days of bed rest (BR10) *A*, a schematic illustrating potential mechanisms associated with increased reactive oxygen species (ROS) production and the proteins investigated in the current study; created in BioRender. *B,C*, expression levels of the fission‐related proteins dynamin‐related protein 1 (DRP1) and mitochondrial fission 1 protein (Fis1). *D‐F*, the expression of mitofusin 1 (MFN1) and optic atrophy 1 (OPA1) (fusion‐related proteins), and phosphorylated DRP1 at serine 637 (DRP1 Ser637), respectively. *G,H*, the expression levels of the mitophagy‐related proteins PTEN‐induced putative kinase 1 (PINK1) and parkin. *I*, the ratio of DRP1 Ser637/DRP1, and *J*, the ratio of OPA1/S‐OPA1. *K*, representative western blots of all proteins analysed. Protein expression levels were normalized to beta‐actin (β‐actin), except for Fis1, which was normalized to total protein content (REVERT). All data were analysed using the Wilcoxon signed‐rank test, with a sample size of *n* = 10, except for Fis1, PINK1 and Parkin where *n* = 9. A Bonferroni correction was applied for nine comparisons. Differences were considered statistically significant when *P* ≤ 0.0056. Effect sizes were calculated using Cohen's *d*.

Our results showed a significant decrease in the total quantity of DRP1 S637 and the ratio of DRP1 S637/DRP1 following 10 days of bed rest (BR10). No significant changes were observed in the levels of MFN1, OPA1 or the L‐OPA1/S‐OPA1 ratio, DRP1 and Fis1, PINK1 and Parkin (Fig. 7). Nevertheless although most of these proteins remained unchanged, the specific reduction in phosphorylated DRP1 at the inhibitory S637 site could suggest a shift in mitochondrial dynamics towards a fission‐prone phenotype.

### Mitochondrial volume density and morphology

As the observed changes in DRP1 S637 and the ratio of DRP1 S637/DRP1 could only suggest a potential shift in mitochondrial phenotype, we assessed mitochondrial morphology using TEM to determine whether shape alterations were present. Additionally given the observed reduction in CS activity, we aimed to directly quantify mitochondrial volume density to provide a definitive, high‐resolution assessment of mitochondrial mass.

**Figure 8:**
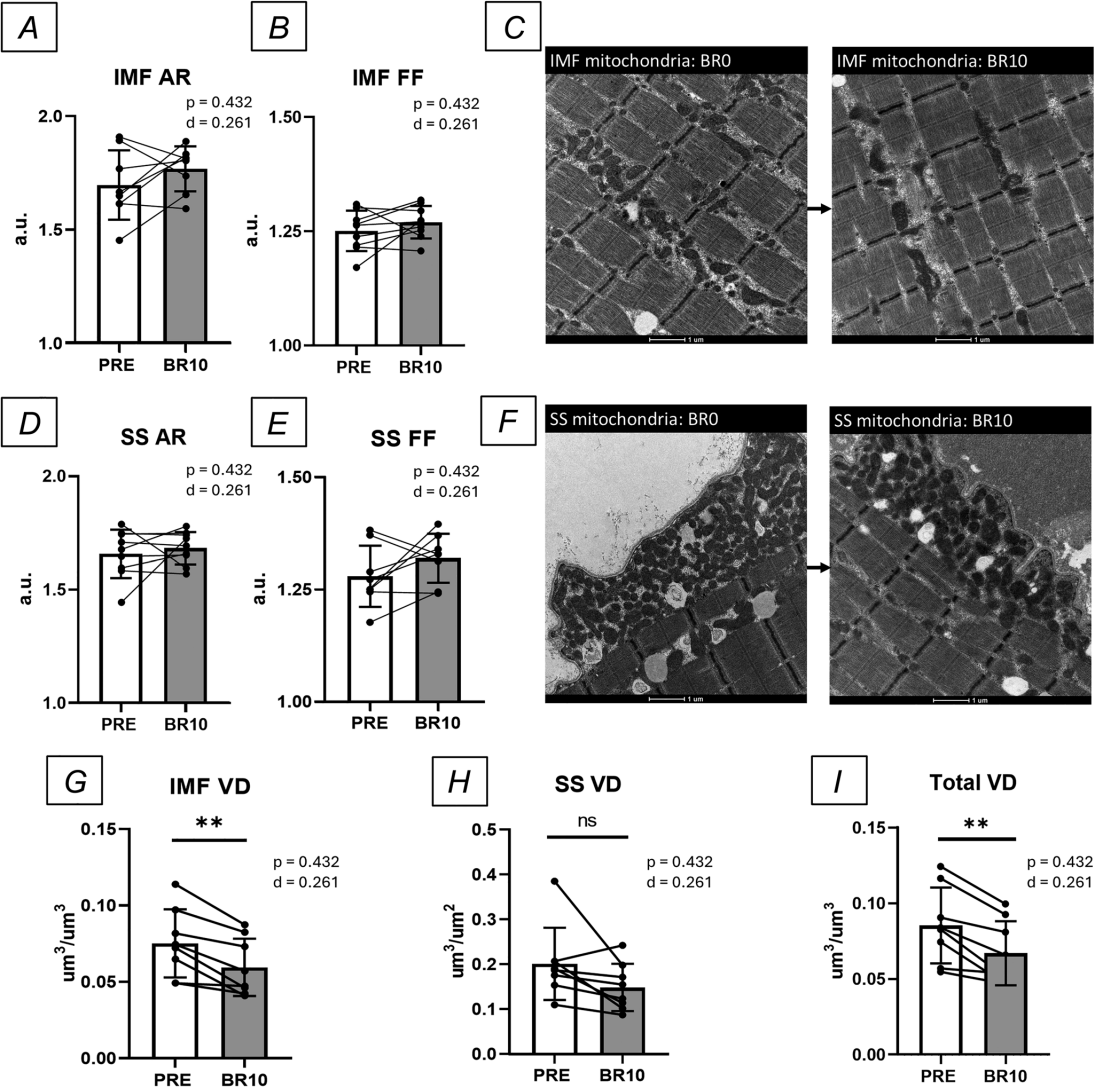
Mitochondrial morphology and volume density assessed before bed rest (PRE) and after 10 days of bed rest (BR10) *A*, the aspect ratio (AR) of intermyofibrillar (IMF) mitochondria; *B*, the form factor (FF) of IMF mitochondria. *D*, the AR of subsarcolemmal (SS) mitochondria; *E*, the FF of SS mitochondria. *C*, representative transmission electron microscopic (TEM) images of IMF mitochondria at PRE and BR10; *F*, representative TEM images of SS mitochondria at PRE and BR10. *G*, the volume density (VD) of IMF mitochondria; *H*, the VD of SS mitochondria; *I*, the Total VD. Data were analysed using the Wilcoxon signed‐rank test, with a sample size of *n* = 8, except for SS AR and SS FF where *n* = 9. ** = *P* ≤ 0.01. Effect sizes were calculated using Cohen's *d*.

As mentioned in the Methods section, we assessed two mitochondrial fractions: intermyofibrillar and subsarcolemmal. Indeed we observed a significant reduction in the mitochondrial volume density of intermyofibrillar mitochondria, and a tendency to volume density reduction in the subsarcolemmal fraction and a significant reduction in mitochondrial total volume density (Fig. 8).

To examine whether the observed protein changes were sufficient to provoke mitochondrial remodelling, 11,596 individual mitochondria were manually traced, including 6076 mitochondria before bed rest (PRE) (IMF = 3191; SS = 2885), and 5520 mitochondria after bed rest (BR10) (IMF = 2834; SS = 2686). On average this corresponded to 399 ± 92 mitochondria analysed per subject for IMF mitochondria and 310 ± 125 mitochondria per subject for SS mitochondria at PRE, and 354 ± 97 mitochondria per subject for IMF mitochondria and 286 ± 91 mitochondria per subject for SS mitochondria at BR10. However we found no significant changes in the key mitochondrial shape descriptors that could suggest ongoing fission or fusion AR and FF (Fig. 8). Together these results show that the reduction in mitochondrial mass was not accompanied by remodelling of their shape.

Additionally, prompted by recent discussions regarding the validity of enzymatic proxies of mitochondrial content, we evaluated the relationship between CS activity and direct structural measures of total mitochondrial volume density (Total VD). Analysis of baseline‐normalised fold changes revealed a weak association between CS activity and Total VD (Pearson's *r* = 0.19, *P* = 0.65). We additionally performed a linear regression analysis examining the relationship between changes (Δ) in CS activity and Total VD. This analysis revealed a moderate positive association (*r* = 0.54, *R* ^2^ = 0.29) that did not reach statistical significance (*P* = 0.17). Taken together these exploratory analyses did not provide strong evidence for a relationship between CS activity and mitochondrial VD in our cohort.

### Transcriptomic adaptations to bed rest

Given the increased maximal ROS production and reduced mitochondrial content observed after 10 days of bed rest in older individuals, we aimed not only to assess whether these phenotypes were reflected at the transcriptional level, but also to explore whether transcriptomic analysis could provide additional insight into other mechanisms involved.

**Figure 9:**
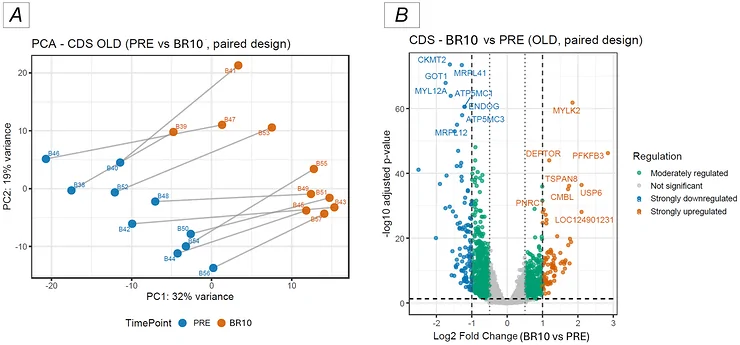
Global sample separation and differential gene expression profiles in older muscle after bed rest *A*, principal component analysis (PCA) plot of variance‑stabilized coding‐sequence (CDS) counts from skeletal muscle of older individuals before (PRE; blue dots) and after 10 days of bed rest (BR10; orange dots). Lines connect paired samples from the same participant (*n* = 10), illustrating coherent within‑subject shifts from PRE to BR10 along the first principal component (PC1). *B*, volcano plot showing differentially expressed genes (DEGs) comparing BR10 *vs*. PRE. The vertical dotted lines indicate |log2 fold change| = 0.5 and 1, used to define moderately (0.5 ≤ |log_2_FC| < 1) and strongly (|log_2_FC| ≥ 1) regulated genes, respectively; the horizontal dashed line indicates the significance threshold (adjusted *P*‑value < 0.05; Wald test with Benjamini‐Hochberg correction). Points are coloured based on regulation category (strongly upregulated, strongly downregulated, moderately regulated or not significant), and representative top transcripts are labelled.

PCA demonstrated a clear separation between the PRE and BR10 timepoints along the first principal component (PC1, 32% of variance), indicating a consistent, coordinated transcriptional response to bed rest across subjects (Fig. 9*A*.

In older skeletal muscle 3217 genes were significantly regulated after 10 days of bed rest (padj < 0.05). Among these 76 genes were classified as strongly upregulated (log_2_ fold change ≥ 1), 113 as strongly downregulated (log_2_ fold change ≤ −1) and 3028 as moderately regulated (0.5 ≤ |log_2_ fold change| < 1) differentially expressed genes (DEGs) (Fig. 9*B*) Top strongly upregulated genes included *MYLK2*, *PFKFB3*, *DEPTOR* and *TSPAN8*, whereas top strongly downregulated genes included *CKMT2*, *GOT1*, *MRRL41*, *MYL12A* and *ATP5MC1* (Fig. 9*B*).

**Figure 10:**
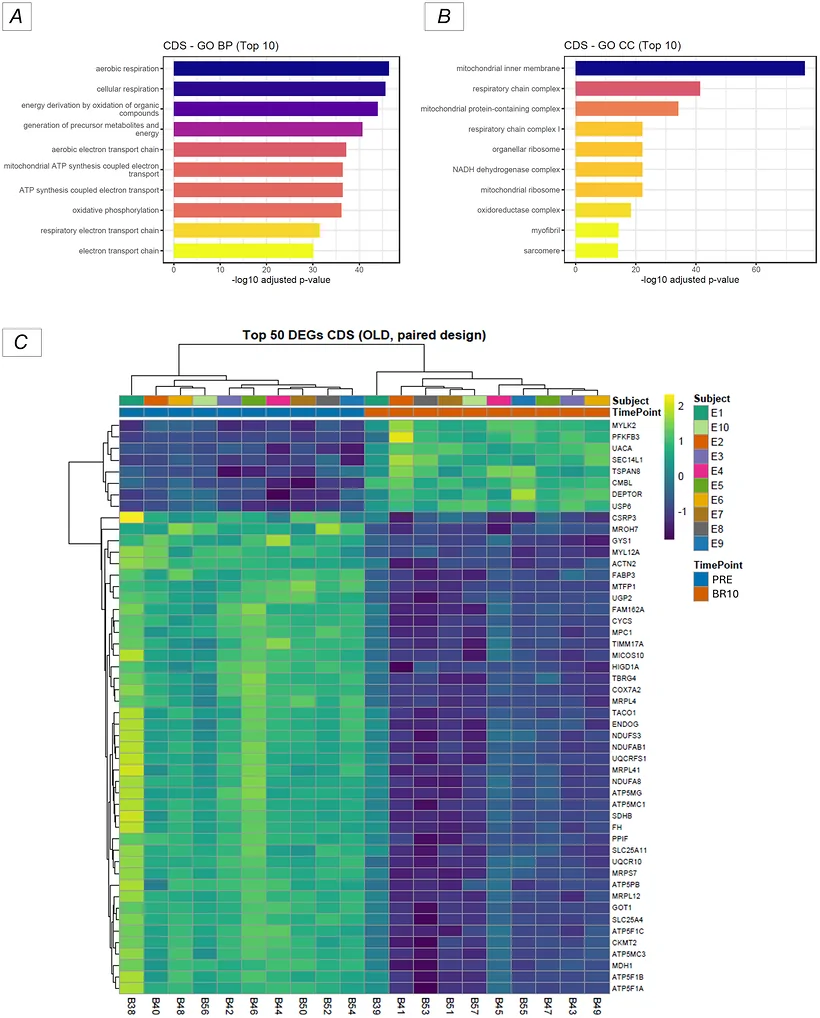
Functional annotation and differential gene expression in older participants after 10 days of bed rest *A*, Gene Ontology Biological Process (GO BP) and *B*, Gene Ontology Cellular Component (GO CC) enrichment analysis showing the top 10 significantly enriched terms among differentially expressed CDS genes (padj < 0.05). Horizontal bars represent –log_10_ adjusted *P*‐values, colour‐coded by significance level. *C*, heatmap of the top 50 differentially expressed genes DEGs (row‐wise *z*‐score of variance‐stabilized counts). Each column represents an individual participant (PRE *vs*. BR10 days of bed rest); colours above the heatmap indicate Subject ID and TimePoint. *N* = 10.

To characterise the functional impact of these changes, GO enrichment analysis was performed for both Biological Processes (GO BP) and Cellular Components (GO CC). The top enriched GO BP pathways were heavily centred on energy metabolism, including ‘aerobic respiration’, ‘cellular respiration’, ‘energy derivation by oxidation of organic compounds’, ‘oxidative phosphorylation’, ‘electron transport chain’ and ATP synthesis‐related processes (Fig. 10*A*). Concurrently GO CC analysis revealed that these transcripts were predominantly localised to mitochondrial sub‐structures, with ‘mitochondrial inner membrane’, ‘respiratory chain complex’ and ‘mitochondrial protein‐containing complex’ emerging as the top three most significantly enriched cellular compartments (Fig. 10*B*). These enrichments were driven by consistent suppression of mitochondrial structural and functional components, as visualised in the top 50 DEGs heatmap (Fig. 10*C*). Specifically we observed downregulation of respiratory chain genes (*CYCS*, *COX7A2*, *UQCR10*), ATP synthase subunits (*ATP5MC1*, *ATP5F1C*, *ATP5PB*, *ATP5MG*, *ATP5F1A*, *ATP5F1B*) and complex I components (*NDUFAB1*, *NDUFS3*, *NDUFA8*). Mitochondrial ribosomal proteins from both the large (*MRPL4*, *MRPL12*, *MRPL41*) and small subunits (*MRPS7*) were reduced, indicating an impairment of mitochondrial translation. Additional downregulated transcripts within this core mitochondrial cluster included genes involved in protein import (*TIMM17A*, *MICOS10*), RNA processing (*TBRG4*), substrate transport (*MPC1*, *SLC25A4*, *SLC25A11*), the TCA cycle (*FH*, *MDH1*, *SDHB*) and energy buffering (*CKMT2*, *GOT1*). Furthermore *PPIF* (encoding Cyclophilin D), a structural component of the mitochondrial permeability transition pore, was significantly downregulated. Conversely bed rest induced a sharp upregulation of a distinct cluster of genes associated with metabolic reprogramming towards glycolysis (*PFKFB3*), cellular stress/apoptosis regulation (*UACA*, *TSPAN8*), deubiquitination (*USP6*) and direct inhibition of mTOR signalling (*DEPTOR*) (Fig. 10*C*).

**Figure 11:**
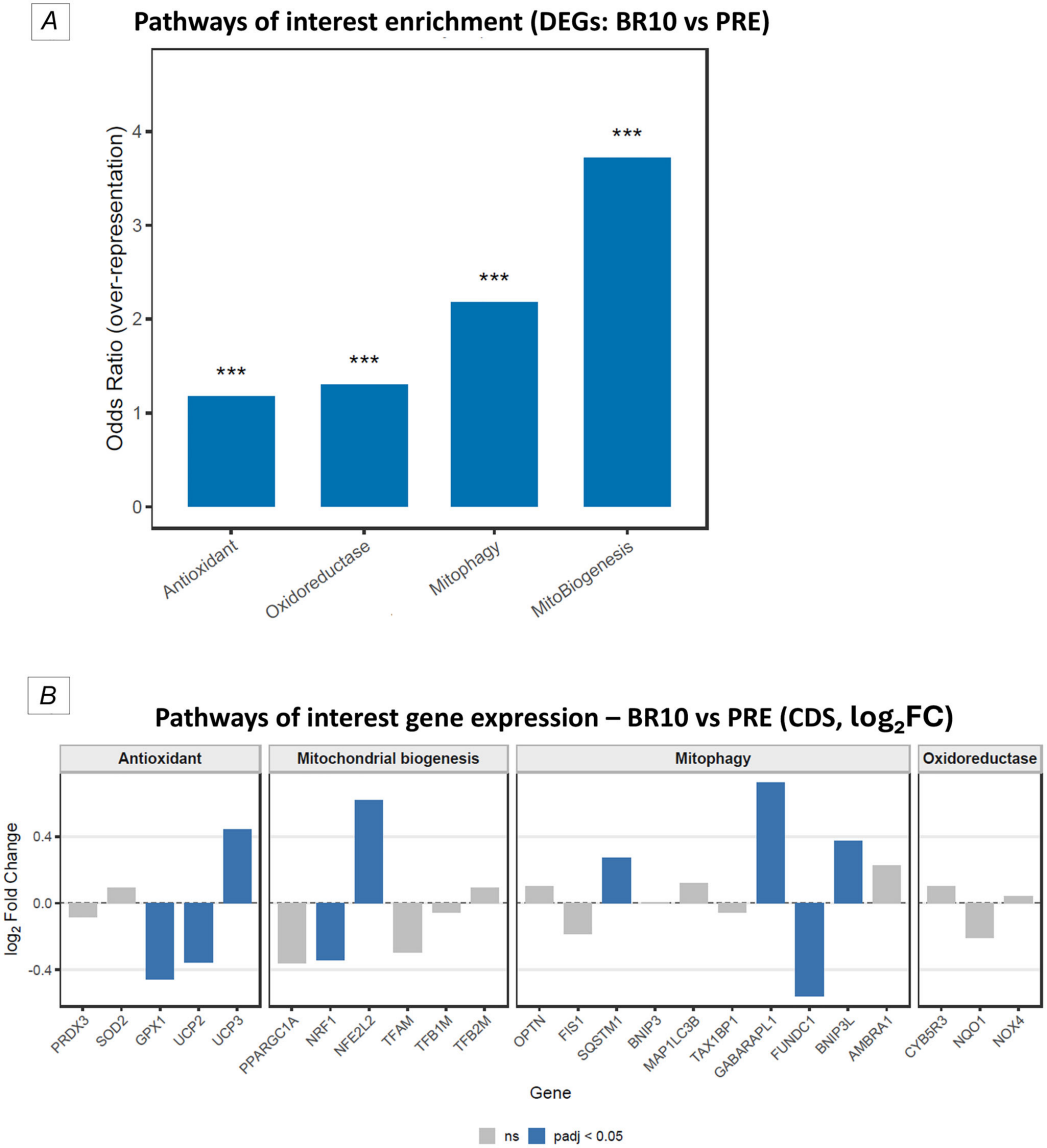
Enrichment of pathways of interest after 10 days of bed rest in older participants *A*, Fisher's exact test of curated pathways related to antioxidant defence, mitochondrial biogenesis, mitophagy and oxidoreductase activity. Horizontal bars represent odds ratios (OR), with values >1 denoting overrepresentation among differentially expressed genes (DEGs). Mitochondrial biogenesis showed the strongest enrichment (pathway size = 277 genes; overlap with DEGs = 145; OR = 3.7; padj = 3.1 × 10^−^^2^ ^5^), followed by mitophagy (93 genes; overlaP = 37; OR = 2.2; padj = 4.8 × 10^−^^4^), oxidoreductase activity (12,197 genes; overlaP = 2930; OR = 1.3; padj = 1.2 × 10^−^^5^) and antioxidant activity (9442 genes; overlaP = 2299; OR = 1.18; padj = 1.5 × 10^−^^4^). Asterisks indicate statistical significance after Benjamini‐Hochberg correction (***padj < 0.001). *B*, expression levels (log_2_ fold change) of constituent genes driving these pathway‐level enrichments. Blue bars highlight individual transcripts reaching statistical significance (padj < 0.05), whereas grey bars represent genes that did not reach statistical significance independently but collectively contributed to pathway‐level overrepresentation. *N* = 10.

Given the functional (elevated ROS) and structural (reduced mitochondrial mass) evidence of early mitochondrial alterations in older muscle after 10 days of bed rest, we next examined transcriptional changes in pathways relevant to mitochondrial quality control and redox regulation. Overrepresentation analysis (Fisher's exact test) revealed significant enrichment of four curated pathways: mitophagy, antioxidant activity, oxidoreductase activity and mitochondrial biogenesis (padj < 0.01; Fig. 11*A*). Mitophagy enrichment was supported by upregulation of *SQSTM1*, *GABARAPL1*, *BNIP3L* (padj < 0.05), together with downregulation of *FUNDC1* (padj < 0.05) and non‐significant downregulation of *FIS 1*. Antioxidant activity enrichment was driven by significant downregulation of *GPX1* and *UCP2* (padj < 0.05) and upregulation of *UCP3* (padj < 0.05; Fig. 11*B*). In the mitochondrial biogenesis pathway although the classic master regulators *PPARGC1A* and *TFAM* displayed minor, non‐significant downward trends, *NRF1* was significantly downregulated (padj < 0.05), whereas *NFE2L2*, which encodes the transcription factor NRF2, was sharply and significantly upregulated (padj < 0.05). Of note the oxidoreductase pathway achieved statistically significant enrichment even though the individual constituent genes, *CYB5R3*, *NQO1* and *NOX4*, did not reach statistical significance independently (padj > 0.05; Fig. 11*B*). This reflects the nature of pathway‐level overrepresentation tests, which evaluate proportional shifts across an entire gene set rather than single‐transcript significance magnitudes. Here a coordinated, modest directional shift across multiple genes collectively yielded a highly significant pathway‐level enrichment signal, even when individual genes remained below the individual statistical threshold.

## Discussion

The present study provides a comprehensive characterisation of mitochondrial adaptations in older adults following 10 days of bed rest, a clinically relevant model of acute hospitalisation and forced immobilisation, integrating functional, morphological, protein‐level and transcriptomic analyses.

Bed rest induced a clear reduction in mitochondrial mass, as evidenced by reduced mitochondrial volume density assessed by electron microscopy, which was accompanied by a decrease in CS activity. It is important to note that mitochondrial volume density and CS activity were assessed in different portions of the same biopsy, which may contribute to some methodological variability between these measures. Notably in our previous study (Zuccarelli et al., 2021), a comparable period of inactivity in young individuals did not alter CS activity. However mitochondrial content was not assessed morphologically in that cohort's thus the maintenance of CS activity was used to suggest that mitochondrial content was maintained in young individuals. Differences in mitochondrial abundance between older and young individuals could reflect an age‐related reduction in skeletal muscle resilience to disuse, with older muscle showing a diminished capacity to maintain mitochondrial content in response to inactivity. Although our exploratory correlation and regression analyses between CS and mitochondrial volume density should be interpreted with caution due to the cohort size, the lack of a strong predictive relationship between them strongly supports our methodological decision to rely primarily on mitochondrial volume density to assess mitochondrial mass, while maintaining CS activity strictly as an enzymatic proxy of mitochondrial content for the normalisation of mitochondrial respiration measurements.

Consistent with the present findings previous studies in older individuals have also reported reduced mitochondrial content following 10 days of bed rest, accompanied by H_2_O_2_ emission (Standley et al., 2020). However a notable discrepancy exists regarding functional respiratory capacity: whereas Standley et al. (2020) observed a significant decline in OXPHOS capacity alongside a decreased abundance of OXPHOS complex proteins, our cohort demonstrated fully preserved respiratory efficiency. In addition in our study OXPHOS complex protein levels were not significantly altered. This apparent discrepancy could be related to the limited sensitivity of western blot−based quantification in detecting modest protein alterations in human skeletal muscle, which is inherently prone to higher biological variability. More fundamentally these conflicting observations underscore that changes in mitochondrial mass, protein content in OXPHOS complexes and actual functional outputs (mitochondrial respiration) do not always occur in a strictly proportional or synchronised manner. Instead short‐term disuse in older muscle may induce a distinct physiological dissociation across different levels of mitochondrial organisation, where reduction in mitochondrial abundance occurs alongside a temporary preservation, or compensatory upregulation, of the intrinsic efficiency of the respiratory machinery.

The reduction in mitochondrial mass observed in the present study is consistent with transcriptomic data indicating an upregulation of mitophagy‐related pathways, pointing towards enhanced mitochondrial removal during short‐term disuse in older muscle. However no changes were detected in canonical mitophagy proteins (PINK1, Parkin) at the protein level despite a reduction in mitochondrial mass, indicating that the contribution of mitophagy cannot be definitively established and warrants further investigation.

Transcriptomic profiling provided broader insights into these pathways. More than 3000 genes were differentially expressed. The transcriptomic response was characterised by a striking convergence between biological processes and cellular components: the downregulation of genes involved in aerobic respiration and oxidative phosphorylation pathways was accompanied by systematic transcriptional suppression of the mitochondrial organelle's structural components. Specifically GO Cellular Component analysis revealed that the most significantly altered transcripts were predominantly localised to the mitochondrial inner membrane, respiratory chain complexes and mitochondrial protein‐containing complexes. Targeted pathway enrichment analysis revealed alterations in mitophagy‐ and oxidative stress‐related mechanisms. Upregulation of cargo adaptors (*SQSTM1, GABARAPL1, BNIP3L*) indicates the activation of components of the autophagic machinery and mitophagy (Di Rienzo et al., 2024; Marinković & Novak, 2021), potentially reflecting an increased demand for mitochondrial turnover during disuse. However this response was accompanied by the downregulation of *FUNDC1*, a receptor that mediates hypoxia‐induced mitophagy and mitochondrial dynamics (Liu et al., 2012), together with a non‐significant reduction in *FIS1*, a component of the mitochondrial fission machinery that contributes to mitochondrial morphology (Pernas & Scorrano, 2016). This combination of increased mitophagy cargo adaptors with reduced expression of mitochondrial fission/fusion regulators is consistent with the impaired mitophagy and imbalance in mitochondrial dynamics − a phenotype usually described in ageing skeletal muscle, where initiation signals are activated but organelle remodelling and downstream clearance steps are impaired (Leduc‐Gaudet et al., 2021; McCormick & Vasilaki, 2018).

Concomitantly mitochondrial ROS production was increased following 10 days of bed rest. Importantly this increase was observed only under non‐phosphorylating conditions, as ADP effectively suppressed ROS production, indicating that the regulatory mechanisms coupling ADP availability to ROS control remained intact. Experimental data addressing redox adaptations to bed rest are still limited. Interestingly similar findings have been reported after 7 days of bed rest in young individuals (Dirks et al., 2020). As recently highlighted by Delfinis et al. (2025), these results suggest that in both cases mitochondria exhibited greater sensitivity to ADP‐mediated attenuation of H_2_O_2_ emission, indicating a specific alteration in how ADP regulates mitochondrial membrane potential. Consistently our observations are in agreement with those of Standley et al. (2020), who reported a trend towards increased H_2_O_2_ emission in nine older adults following 10 days of bed rest under non‐phosphorylating conditions. The higher mitochondrial ROS production observed under non‐phosphorylating conditions, together with the unchanged IC_50_ following 10 days of bed rest, may suggest a greater inhibitory control of ROS emission (i.e. unchanged H_2_O_2_ release at given ADP concentrations) after bed rest. This could potentially reflect a compensatory response that may partially mitigate a disuse‐induced redox imbalance.

In parallel targeted transcriptomic analyses revealed a significant enrichment of antioxidant and oxidoreductase pathways, exposing a highly discordant molecular response to inactivity and suggesting a potential dysregulation of ROS buffering capacity. On one hand frontline cellular defence was severely compromised, as evidenced by significant downregulation of *GPX1*, which directly coordinates peroxide clearance (Lubos et al., 2011), alongside the significant downregulation of *UCP2*. On the other hand the muscle‐specific *UCP3* was strongly and significantly upregulated. This prominent elevation of *UCP3* represents a targeted compensatory attempt to induce mild mitochondrial proton leak, a well‐established physiological mechanism deployed to lower mitochondrial membrane potential and lower the driving force for further superoxide production under metabolic stress conditions (Garvey, 2003; Toime & Brand, 2010). Although these changes may contribute to the observed increase in mitochondrial ROS emission, the relative contribution of altered ROS clearance *versus* increased production cannot be definitively resolved, as these observations are limited to the transcript level. Future studies integrating protein‐level analyses will be required to confirm these mechanisms.

Overall these alterations align with the functional evidence of increased ROS emission and suggest a disrupted redox equilibrium and compromised ROS clearance. Rather than a coordinated stress‐adaptation programme, the transcriptomic signature suggests insufficient compensatory response of the older skeletal muscle to the oxidative stress. In line with this interpretation no changes were detected in OXPHOS complexes’ proteins or respiratory supercomplex abundance, arguing against structural damage of the respiratory machinery as the primary driver of increased ROS production.

Focusing on mitochondrial morphology we observed decreased phosphorylation of DRP1 at Ser637 that suggested a potential shift towards mitochondrial fragmentation. However this molecular change was not accompanied by detectable alterations in individual mitochondrial shape descriptors across either SS or IMF fractions, with both AR (an index of organelle elongation) and FF (a measure of geometric branching and network complexity) (Picard et al., 2013) remaining unchanged after bed rest. Thus TEM data indicate the preservation of mitochondrial morphology at the level of individual organelle geometry despite the reduction in mitochondrial content.

Consistent with our interpretation prolonged periods of disuse have been shown to induce clear changes in mitochondrial shape descriptors (Eggelbusch et al., 2024), suggesting that longer inactivity may be required for these early molecular signals to manifest as morphological adaptations. Of note the impairment of mitochondrial structure and function with ageing overall appears to be less consistent than commonly assumed, as available data on mitochondrial biogenesis and dynamics in humans are limited and often contradictory. Indeed studies assessing markers such as citrate synthase (CS) activity, PGC‐1α mRNA and protein levels, NRF1 and TFAM protein expression, as well as mitochondrial dynamics regulators including MFN2 and DRP1, report both reductions and preservation, or even upregulation, of these parameters, possibly reflecting compensatory mechanisms (Rossi et al., 2026). Our transcriptomic data mirror this complexity, capturing a discordant response within the ‘mitochondrial biogenesis’ pathway. Classic master regulators like *PPARGC1A* (PGC‐1α) and *TFAM* were downregulated (non‐significantly), alongside significant suppression of *NRF1* expression. Crucially this was counterbalanced by a significant upregulation of *NFE2L2*, which encodes the transcription factor NRF2, a central regulator of antioxidant and cytoprotective responses (Ngo & Duennwald, 2022). This implies that although baseline organelle biogenesis signalling is transcriptionally downregulated, the muscle activates an emergency cytoprotective gene programme (*NFE2L2*) in response to the early oxidative stress of disuse.

Importantly although numerous oxidative phosphorylation genes were transcriptionally suppressed, protein‐level analyses of proteins of representative OXPHOS complexes did not reveal impairments, consistent with preserved functional capacity. A similar discrepancy was observed for mitophagy regulators: despite enrichment of mitophagy‐related transcripts, no changes were detected in PINK1 and Parkin protein levels. Such transcript‐protein mismatches may reflect both methodological coverage (RNA sequencing captured the full range of coding sequences, whereas western blotting targeted only a limited subset of proteins) and biological factors (as many mitochondrial proteins are relatively long‐lived and not immediately degraded when transcript levels decline). In support of this notion recent high‐resolution in vivo mass spectrometry tracking in rodents has revealed that many structural components of the respiratory chain complexes and the inner mitochondrial membrane possess remarkably long half‐lives, extending up to several weeks in mature tissues (Gugel et al., 2026), which could explain why a 10‐day transcriptional downregulation did not deplete the pre‐existing pool of OXPHOS proteins. Conversely paradigms outlined by Hood (2001) indicate that certain mitochondrial proteins exhibit relatively short half‐lives of approximately 1 week, allowing for rapid proteomic and enzymatic adaptations following structural or behavioural interventions. This capacity for accelerated remodelling is supported by the findings of Standley et al. (2020), who observed significant declines in OXPHOS protein abundance within a matching 10‐day bed rest timeframe. A comparable phenomenon was reported in 3‐day disuse study in women, where OXPHOS‐related genes were downregulated without detectable OXPHOS complexes’ protein content alteration (Popov et al., 2023). Alternatively these results could suggest that transcriptomic changes precede functional impairments of mitochondrial respiration, with protein abundance of OXPHOS complexes preserved during the earlier stages of inactivity. More broadly these findings should be interpreted within the temporal hierarchy of disuse adaptations, whereby rapid transcriptomic reprogramming may anticipate proteomic remodelling that unfolds over longer timescales, whereas structural and functional declines reflect cumulative changes in protein content and function that may not yet be detectable at early time points.

Despite reduced mitochondrial mass and increased ROS emission, mitochondrial respiratory function was preserved. At the functional level high‐resolution respirometry demonstrated maintained submaximal and maximal OXPHOS and electron transport system (ETS) capacities following bed rest, together with unaltered ADP sensitivity across physiological ranges. Notably a previous study by our group showed that a comparable period of inactivity in young individuals also did not alter OXPHOS efficiency (Zuccarelli et al., 2021), suggesting that preserved respiration is a common response to 10‐day bed rest in both young and older muscle. These findings indicate that mitochondrial respiratory efficiency is resilient to short‐term inactivity in ageing muscle. The absence of changes in cyclophilin D and the truncated Cyclophilin D (ΔN‐CyPD) following bed rest supports the maintenance of the functional integrity of mitochondria. Several studies show that aged mitochondria are more prone to PTP opening, a trait also evident in human skeletal muscle, where, differently from other tissues such as heart, it appears as an intrinsic ageing characteristic, unaffected by exercise (Gouspillou et al., 2014). Our finding of unchanged levels of both CyPD forms may suggest that PTP opening propensity remained unchanged after bed rest, in accordance with the preserved mitochondrial bioenergetics and morphology, although occurrence of post‐translational modifications of CyPD able to sensitise PTP cannot be excluded (Coluccino et al., 2024). Interestingly although CyPD protein levels were unchanged, downregulation of its transcript, *PPIF*, may represent an early molecular signature of a shift in PTP regulation that has not yet manifested at the protein level.

Notably when respiratory capacity was normalized to mitochondrial content, OXPHOS/CS was significantly increased after bed rest. Thus mitochondrial respiration not only failed to decline but was actually increased after bed rest, indicating enhanced intrinsic mitochondrial function. Even considering the limitations of CS activity as an indirect marker of mitochondrial content, this may suggest that, in the context of disuse‐induced mitochondrial loss, ageing mitochondria mount an effective compensatory response by intrinsically increasing respiratory function (i.e. OXPHOS/CS) to preserve overall oxidative capacity. The upregulated oxygen consumption aligns with the preserved mitochondrial respiration and the reduced citrate synthase activity observed after 4 days of bed rest in young participants (Larsen et al., 2018). This adaptive mechanism appears remarkable but may be transient. Indeed prolonged inactivity likely overwhelms this compensation, as demonstrated by Eggelbusch et al. (2024), who reported reduced mitochondrial respiration following 55 days of bed rest in young individuals.

Finally these findings seem to indicate that, in older adults, the primary limitations to oxidative metabolism following short‐term disuse are unlikely to reside at the level of intrinsic mitochondrial respiration. When considering oxidative metabolism at the whole‐body level during maximal exercise (i.e. $\dot{V}O_{2}\max$), rather impairments in cardiovascular function, microvascular and endothelial regulation and peripheral oxygen diffusion may represent the predominant determinants of the observed decline in oxidative capacity.

## Limitations and future directions

Although the number of participants was relatively limited, this should be interpreted in the context of the logistical, economic and ethical constraints inherent to bed rest studies. Such campaigns require extensive resources, including high costs and the allocation of an entire hospital ward for several weeks, which intrinsically limits sample size. These constraints are further amplified in studies involving older individuals, who are exposed to increased risks of cardiovascular, cerebrovascular and metabolic complications that are closely scrutinized by ethical committees. Notably several recent and well‐established bed rest studies (Eggelbusch et al., 2024; Larsen et al., 2018; Zuccarelli, De Martino et al., 2025) have successfully employed comparable sample sizes (n = 8−10), supporting the adequacy of the present cohort.

Although the integration of functional and transcriptomic analyses represents a strength of this study, protein‐level analyses were necessarily limited to a small set of markers, potentially overlooking broader proteomic adaptations, particularly in antioxidant defence and mitophagy pathways. In addition transcriptomic profiling was performed at a single post‐intervention time point (10 days), preventing insights into the early temporal dynamics of mitochondrial responses to disuse. Finally the findings are restricted to older male participants, as the inclusion of older women is ethically challenging due to their greater susceptibility to disuse‐induced atrophy and frailty. Future studies incorporating multiple time points, broader molecular profiling and more diverse cohorts will further advance the understanding of mitochondrial adaptations to inactivity. Beyond the adaptations driving skeletal muscle deconditioning, the temporal dynamics of mitochondrial recovery following the cessation of bed rest remain incompletely characterized. Although short‐term disuse rapidly induces mitochondrial abundance reduction and redox stress in skeletal muscle, recovery of mitochondrial content and function is known to depend on coordinated activation of biogenesis and quality control pathways, yet the extent and time course of full reversibility remain unclear (Memme et al., 2021). Importantly mitochondrial adaptations to reloading or exercise may not directly mirror those observed during disuse, suggesting that recovery represents a distinct biological process rather than a simple reversal of inactivity‐induced alterations (Von Ruff et al., 2025). Future studies incorporating muscle biopsies during retraining phases, as well as controlled animal models such as hindlimb suspension followed by recovery, will be essential to define the temporal trajectory of mitochondrial structural and functional restoration and to determine the contributions of mitochondrial biogenesis, mitophagy and network remodelling.

In conclusion, functionally we observed an increase in ROS emission in older muscle after 10 days of bed rest, occurring upstream of any detectable impairment in OXPHOS capacity. This was accompanied by a reduction in mitochondrial mass, which was transcriptionally linked to enrichment of mitophagy pathways. At the same time transcriptomic analysis revealed alterations in antioxidant and oxidoreductase regulators, indicating a potential dysregulation in ROS clearance capacity. Notably the reduction in mitochondrial abundance was transcriptionally associated with mitophagy pathway activation. This supports the notion of dysregulated mitochondrial turnover as a likely contributor to decreased mitochondrial content.

## Additional information

## Competing interests

None declared.

## Author contributions

M.V.N., B.G., R.P., B.S.: Conceptualisation. B.S., E.M., L.Z., E.L., S.A., L.B., M.G., G.B., C.G., G.L.: Methodology. E.M., L.Z., E.L., S.A., L.B., C.G., G.L.: Data analysis. E.M. and L.Z.: Writing − original draft. M.V.N., B.G., E.M., L.Z., E.L., S.A., L.B., R.B., M.A.P., M.P., O.R., R.P., B.S., M.G., G.B., C.G., G.L.: Writing − review and editing. M.V.N., B.G., B.S., R.P.: Project administration. M.V.N., B.G., R.P., B.S., E.M.: Funding acquisition. M.V.N., B.G., B.S., R.P.: Resources. M.V.N., B.G.: Supervision. All authors reviewed, revised and approved the final version of the manuscript. They also agreed to take full responsibility for all aspects of the work, ensuring that any questions regarding its accuracy or integrity are thoroughly investigated and addressed.

## Funding

The present study was funded by the PRIN project (‘InactivAge’ n. 2020EM9A8X) to M.V.N. and B.G. We also acknowledge co‐funding from Next Generation EU to M.V.N. in the context of the National Recovery and Resilience Plan, Investment PE8 − Project Age‐It: ‘Ageing Well in an Ageing Society’. This work has received funding from the European Union's Horizon 2020 research and innovation programme under the Marie Skłodowska‐Curie grant agreement 101034319 and from the European Union − NextGeneration EU. This study was also co‐funded by the ARIS (‘J5‐4593’). The views and opinions expressed are only those of the authors and do not necessarily reflect those of the European Union or the European Commission. Neither the European Union nor the European Commission can be held responsible for them.

## Generative AI statement

No generative artificial intelligence tools were used in the preparation of this manuscript.

## Supporting information


Peer Review History

## Data Availability

The collection of TEM images acquired in this study is openly available and can be accessed at osf.io/jvk4g. The raw RNA‐sequencing data (FASTQ files) generated in this study are publicly available at osf.io/8ahzu. The other data that support the findings of this study will be made available from the corresponding authors upon reasonable request.
