# Supplementary material for: Preserved mitochondrial respiration in presence of oxidative stress and reduced mitochondrial mass after 10‐day bed rest in older adults
Source: J Physiol. 2026 Sep 11;604(18):7772–98. doi: 10.1113/JP291588 (PMC13577690; doi:10.1113/JP291588)
Supplement: Supplementary file 1 [file TJP-604-7772-s001.pdf]

The Journal of Physiology

Preserved mitochondrial respiration in the presence of oxidative stress and reduced mitochondrial mass after 10-day bed rest in older adults

Evgeniia Motanova, Lucrezia Zuccarelli, Evgenii Lysenko, Stefano Amoretti, Marco Pirazzini, Ornella Rossetto, Lorenza Brocca, Roberto Bottinelli, Maria Antonietta Pellegrino, Giovanni Baldassarre, Clarissa Gissi, Giovanna Lippe, Mladen Gasparini, Bostjan Simunic, Rado Pisot, Bruno Grassi, and Marco Narici
DOI: 10.1113/JP291588

Corresponding author(s): Evgeniia Motanova (evgeniia.motanova@unipd.it)

The following individual(s) involved in review of this submission have agreed to reveal their identity: Casper Soendenbroe (Referee #1)

Review Timeline:

Submission Date:	17-Apr-2026
Editorial Decision:	03-Jun-2026
Revision Received:	30-Jun-2026
Accepted:	10-Aug-2026

Senior Editor: Karyn Hamilton

Reviewing Editor: Russell Hepple

Transaction Report:

(Note: With the exception of the correction of typographical or spelling errors that could be a source of ambiguity, letters and reports are not edited. Depending on transfer agreements, referee reports obtained elsewhere may or may not be included in this compilation. Referee reports are anonymous unless the Referee chooses to sign their reports.)

Dear Dr Motanova,

Re: JP-RP-2026-291588 "**Preserved mitochondrial respiration in the presence of oxidative stress and reduced mitochondrial content after 10-day bed rest in older adults**" by Evgeniia Motanova, Lucrezia Zuccarelli, Evgenii Lysenko, Stefano Amoretti, Marco Pirazzini, Ornella Rossetto, Lorenza Brocca, Roberto Bottinelli, Maria Antonietta Pellegrino, Giovanni Baldassarre, Clarissa Gissi, Giovanna Lippe, Mladen Gasparini, Bostjan Simunic, Rado Pisot, Bruno Grassi, and Marco Narici

Thank you for submitting your manuscript to The Journal of Physiology. It has been assessed by a Reviewing Editor and by 2 expert referees and we are pleased to tell you that it is acceptable for publication following satisfactory revision.

The referee reports are copied at the end of this email.

Please address all the points raised and incorporate all requested revisions or explain in your Response to Referees why a change has not been made. We hope you will find the comments helpful and that you will be able to return your revised manuscript within 4 weeks. If you require longer than this, please contact journal staff: jp@physoc.org.

Your revised manuscript should be submitted online using the link in your Author Tasks Link Not Available. This link is accessible via your account as Corresponding Author; it is not available to your co-authors. If this presents a problem, please contact journal staff (jp@physoc.org). Image files from the previous version are retained on the system. Please ensure you replace or remove any files that are being revised.

If you do not wish to submit a revised version of your manuscript, you must inform our journal staff (jp@physoc.org) or reply to this email to request withdrawal. Please note that a manuscript must be formally withdrawn from the peer review process at one journal before it may be submitted to another journal.

TRANSPARENT PEER REVIEW POLICY: To improve the transparency of its peer review process The Journal of Physiology publishes online, as supporting information, the peer review history of all articles accepted for publication. Readers will have access to decision letters, including Editors' comments and referee reports, for each version of the manuscript, as well as any author responses to peer review comments. Referees can decide whether or not they wish to be named on the peer review history document.

ABSTRACT FIGURES: Authors are expected to use The Journal's premium BioRender account to create/redraw their Abstract Figures. Information on how to access this account is here: https://physoc.onlinelibrary.wiley.com/journal/14697793/biorender-access.

This will enable Authors to create and download high-resolution figures. If authors have used the free BioRender service, they can use the instructions provided in the link above to download a high-resolution version suitable for publication.

The link provided should only be used for the purposes of this submission. Authors will be charged for figures created on this account if they are not related to this manuscript submission.

LANGUAGE EDITING AND SUPPORT FOR PUBLICATION: If you would like help with English language editing, or other article preparation support, Wiley Editing Services offers expert help, including English Language Editing, as well as translation, manuscript formatting, and figure formatting at www.wileyauthors.com/eeo/preparation. You can also find resources for Preparing Your Article for general guidance about writing and preparing your manuscript at www.wileyauthors.com/eeo/prepresources.

RIGOUR AND REPRODUCIBILITY: We recommend authors consult The Journal's Rigour and Reproducibility requirements to ensure that their manuscript is suitable for publication: https://physoc.onlinelibrary.wiley.com/pb-assets/hub-assets/physoc/documents/TJP-Rigour-and-Reproducibility-Requirements.pdf

REVISION CHECKLIST:

Check that your Methods section conforms to journal policy: https://jp.msubmit.net/cgi-bin/main.plex?form_type=display_requirements#methods

Check that data presented conforms to the statistics policy: https://jp.msubmit.net/cgi-bin/main.plex?form_type=display_requirements#statistics

Upload a full Response to Referees file. To create your 'Response to Referees' copy all the reports, including any comments from the Senior and Reviewing Editors, into a Microsoft Word, or similar, file and respond to each point, using font or background colour to distinguish comments and responses and upload as the required file type.

Please upload two versions of your manuscript text: one with all relevant changes highlighted and one clean version with no changes tracked. The manuscript file should include all tables and figure legends, but each figure/graph should be uploaded

as separate, high-resolution files. The journal is now integrated with Wiley's Image Checking service. For further details, see: https://www.wiley.com/en-us/network/publishing/research-publishing/trending-stories/upholding-image-integrity-wileys-image-screening-service

You may also upload:

- 'Potential Cover Art' for consideration as the issue's cover image
- Appropriate Supporting Information (Video, audio or data set: see https://jp.msubmit.net/cgi-bin/main.plex?form_type=display_requirements#supp).

We look forward to receiving your revised submission.

If you have any queries, please reply to this email and we will be pleased to advise.

Yours sincerely,

Karyn Hamilton
Senior Editor
The Journal of Physiology

REQUIRED ITEMS

- Author photo and profile. First or joint first authors are asked to provide a short biography (no more than 100 words for one author or 150 words in total for joint first authors) and a portrait photograph. These should be uploaded and clearly labelled together in a Word document with the revised version of the manuscript. See Information for Authors for further details.

- You must upload original, uncropped western blot/gel images (including controls) if they are not included in the manuscript. This is to confirm that no inappropriate, unethical or misleading image manipulation has occurred. These should be uploaded as 'Supporting information for review process only'. Please label/highlight the original gels so that we can clearly see which sections/lanes have been used in the manuscript figures. For more information, see: https://physoc.onlinelibrary.wiley.com/hub/journal-policies#imagmanip.

- Papers must comply with the Statistics Policy: https://jp.msubmit.net/cgi-bin/main.plex?form_type=display_requirements#statistics.

In summary:

- If n {less than or equal to} 30, all data points must be plotted in the figure in a way that reveals their range and distribution. A bar graph with data points overlaid, a box and whisker plot or a violin plot (preferably with data points included) are acceptable formats.

- If n > 30, then the entire raw dataset must be made available either as supporting information, or hosted on a not-for-profit repository, e.g. FigShare, with access details provided in the manuscript.

- 'n' clearly defined (e.g. x cells from y slices in z animals) in the Methods. Authors should be mindful of pseudoreplication.

- All relevant 'n' values must be clearly stated in the main text, figures and tables.

- The most appropriate summary statistic (e.g. mean or median and standard deviation) must be used. Standard Error of the Mean (SEM) alone is not permitted.

- Exact p values must be stated. Authors must not use 'greater than' or 'less than'. Exact p values must be stated to three significant figures even when 'no statistical significance' is claimed.

- Please include an Abstract Figure file and an Abstract Figure legend. An appropriate figure legend, which should not exceed 150 words in length, should be included in the main manuscript file. The Abstract Figure is a piece of artwork designed to give readers an immediate understanding of the research and should summarise the main conclusions. If possible, the image should be easily 'readable' from left to right or top to bottom. It should show the physiological relevance of the manuscript so readers can assess the importance and content of its findings. Abstract Figures should not merely recapitulate other figures in the manuscript. Please try to keep the diagram as simple as possible and without superfluous information that may distract from the main conclusion(s). Abstract Figures must be provided by authors no later than the revised manuscript stage and should be uploaded as a separate file during online submission labelled as File Type 'Abstract Figure'. Please also ensure that you include the figure legend in the main article file. All Abstract Figures should be created

using BioRender. Authors should use The Journal's premium BioRender account to export high-resolution images. Details on how to use and access the premium account are included as part of this email.

- New: Authors are required to fully disclose the use of any Generative AI (GAI) tools used in the preparation of their manuscript. This disclosure should also be included in the 'Methods' section (or a suitable location for article types not requiring Methods), and include the name(s), model and version of AI technology used, and exact details on the purpose for, and methods of, its use.

If no AI tool has been used, this should also be specifically stated within the manuscript.

EDITOR COMMENTS

Reviewing Editor:

Thank you for submitting your work to The Journal of Physiology. Two reviewers have provided a thorough review and although they were enthusiastic about the merits of the work, both sought additional clarification and revision. With respect to reviewer 2's questions about the use of CS activity to normalize the respirometry rather than EM-determined mitochondrial volume density, I would suggest that you specify in the Discussion the rationale for this was that there could be disparity between the EM-based mitochondrial volume density and the CS activity in the muscle bundle since the measures were performed on separate pieces of muscle from the same biopsy. You should also consider addressing the potential limitations of using CS activity as an index of mitochondrial content in this same section how this might impact the interpretation of elevated mitochondrial respiration per unit of mitochondrial volume.

Senior Editor:

Thank you for submitting your manuscript for consideration by The Journal of Physiology. As part of the peer review process, we recruited two Referees with expertise in this field of study. Both referees noted that there is potential for your manuscript to be influential in this field of study, however they also both noted some concerns that would need to be satisfactorily addressed for this impact to be realized. At this point we would like to invite you to respond point-by-point to each Referee's concerns, making corresponding revisions to your manuscript. We look forward to seeing your revised work and appreciate your interest in The Journal of Physiology!

Thank you for your attention to The Journal's statistical reporting policy. Could you please revisit Figure 11A to provide precise p-values as you have with the other figures? Thank you.

REFEREE COMMENTS

Referee #1:

Dear Motanova et al.,

I have evaluated your manuscript and find it clear, well written, and potentially important. Below are my comments for your consideration.

Major comments

1. I remain skeptical about the use of phrases such as "the combined effects of ageing and disuse" and "their combined effects on...". To me, this implies an interaction between ageing and disuse. However, the study assess the effects of disuse in older adults, not an ageing × disuse interaction. This distinction is important and should be made clear throughout the manuscript.

2. It is my understanding that the project on which this manuscript is based has also been used for other publications. This is of course acceptable, but should be handled transparently. Please clarify whether data from the same participants have been published previously, and if so, where.

3. Related to this, was muscle atrophy actually induced by the bed-rest model? In other words, did the model work as intended? This should either be shown in the present manuscript or clearly referenced if already reported elsewhere.

4. If BR0 and BR10 RNA-seq samples are paired within individuals, the differential expression model should account for participant identity. A model including only Time treats observations as independent and may fail to account for substantial inter-individual baseline variation.

5. Citrate synthase activity and EM data suggest lower mitochondrial content, whereas Complex I-V protein abundance normalized to β-actin appears unchanged. Please discuss this apparent discrepancy. Does this indicate preserved OXPHOS protein abundance despite lower mitochondrial content, altered mitochondrial composition, or possible limitations in the sensitivity of the protein measurements?

6. For Table 7/Figure 7, please clarify whether p-values were adjusted for multiple comparisons across the mitochondrial protein panel. If not, please apply an appropriate correction or clearly state that these analyses are exploratory.

7. I find the correlation analysis between mitochondrial fusion/fission proteins and ROS production problematic as currently presented. The more correlations that are tested, the greater the chance of finding a significant one. If the strongest correlation as shown is borderline significant and no correction for multiple testing has been applied, the evidence for a robust link is weak. If you choose to keep this analysis, please state explicitly that it is exploratory and report exactly how many correlations were tested. A summary table of all correlations may also be useful.

8. Please clarify whether the GO-CC analysis, which was mentioned in methods, provided any information related to mitochondrial number, composition, or mitochondrial compartments.

Specific comments

- Is there a particular reason why p-values and effect sizes are inserted in tables above each figure? These could perhaps be shown directly in the corresponding figure, where relevant.

- In general, mitochondrial biology papers contain many abbreviations. I recommend defining the relevant abbreviations in each figure legend to improve readability.

- Figure 2 and others: The subfigure numbering should be consistent throughout the manuscript. Please use one system.

- Figure 6A: Please examine the clear outlier. The value appears approximately 7-8-fold higher than all others, raising the possibility of a methodological issue. If retained, please justify its inclusion; alternatively, consider presenting analysis with and without this value.

- Line 106-107: "As the primary site of energy production within muscle cells..." This is too broad. Mitochondria are the primary site of oxidative ATP production, but not all energy production.

- Line 155-156: "Ten older males (mean age: 68.5 {plus minus} 2.64 years; height: 172.7 {plus minus} 6.31 cm; body mass: 85.6 {plus minus} 12.25 kg..." Please adjust decimals to a level that reflects realistic measurement precision.

- Line 175: "the ambulatory period" is unclear in this context. Please define.

- Line 180-181: "...from contralateral legs before (PRE) and after (BR10) the bed rest intervention." It is not clear what "contralateral" refers to here.

- Line 508-511: The reported p-values reported in text do not appear to correspond to the values shown in Table 3. Please clarify which comparisons these p-values refer to.

- Line 644-646: The total number of counted mitochondria is less informative than the number of mitochondria analysed per subject/sample. Please provide the latter.

- Line 695: Please define what is meant by "strongly downregulated", "strongly upregulated", and "moderately regulated" in this context.

Referee #2:

Motanova and colleagues present a thorough investigation into the effects of 10 days of bed rest in older adults on mitochondrial respiration, volume, and morphology. The authors report that while 10-days of bed rest had no effect on respiratory capacity, there was an increase in ROS emission. The authors also share data that suggest that mitochondrial protein complex abundance was unchanged as was supercomplex formation. The authors suggest that modulation in ROS emission may have been facilitated by increased mitochondrial fission as measured by markers of mitochondrial dynamics and a decrease in mitochondria (presumably by increased mitophagy) as measured by TEM. Finally, the others present

transcriptomic data that suggests that 10 days of bed rest led to mitochondrial remodeling, increasing expression of genes related to redox and mitophagic pathways. Overall, the study presented is very well-done and the findings are interesting. A similar bed-rest model has been employed in adults prior by a different group. These data seem to conflict with some of their findings (present study does not report a decline in respiratory capacity, the prior study did). It would seem appropriate to acknowledge this study to greater extent. Otherwise, there are no major concerns, only some suggestions in hopes to improve the manuscript. This is an otherwise well-written and well-presented study.

It is odd the authors rely on citrate synthase activity as a metric for mitochondrial mass when the authors have gone through the very rigorous process of measuring mitochondrial density. Previous papers have demonstrated that citrate synthase might be moderately correlated with citrate synthase but is poor at predicting changes in mitochondrial volume (28580772). Thus, using citrate synthase activity as the indicator that mass changed is odd (example: line 754)

For that reason, the authors ought to consider expressing mitochondrial respiration relative to muscle mass and mitochondria mass/density instead of citrate synthase activity.

Additionally, out of the sake of rigor and continuing to evaluate the utility of citrate synthase as a marker of mitochondrial mass/density, would the authors consider checking if:

1. Citrate synthase activity correlates with measures of mitochondrial density?

2. Changes in CS activity predicts changes in changes in mitochondrial density?

Members of the study team have previous published a technique of investigating mitochondrial ADP sensitivity using a two-phase approach in the young bed-rest cohort (40285367). Why did the authors not employ that approach on these data?

The interrogation of mitochondrial fission and fusion may be strengthened if the authors published more TEM data. Is it possible to report average surface area/volume of mitochondria pre and post bed rest? Presumably, increased fission and decreased fusion would drive for smaller sized mitochondria lending to a more fragmented phenotype.

Line 764: "...no changes were detected in canonical mitophagy proteins... indicating that contribution of mitophagy cannot be definitely established..." did the authors probe for protein expression related to mitochondrial biogenesis?

It is interesting that despite a decrease in mitochondrial volume, there was no change in mitochondrial OXPHOS complex protein expression. To one extent, this may suggest that only certainly mitochondrial proteins (unrelated to OXPHOS) are targeted for degradation and/or the matrix shrinks in size.

Line 875: the authors suggest that respiration was enhanced on a "per-mitochondrion" basis. However, the authors did not count the number of mitochondria. Rather, they provide mitochondrial mass/volume.

Line 890: the authors indicate that central factors may determine decline in oxidative capacity. Are they referring to oxidative capacity at the mitochondrial, muscle, or human-level? On that note, did the authors evaluate any physical outcomes (VO2max, DXA, muscle volume, strength)?

This was a resource intensive study. Understandably, it is unlikely that there will be more data coming from this study. However, it would be very interesting to understand how mitochondria recover after bed-rest is discontinued and normal activity is resumed. Understanding the recovery (or lack thereof) seems just as important to understand.

Minor comments

Line 387: it is a significant undertaking to manually trace mitochondria. Was this done by one individual? If done by more than 1 individual, was there any internal verification/inter-rater tests done to ensure no bias? Were these individuals blinded to samples? Were the number of mitochondria identified similar from participant to participant?

Figure 8 and Table 8: will the authors consider not using abbreviations for the graphs and if possible, also for the table?

Line 803: the authors reference "subtle remodeling of cellular redox regulation" could they be more explicit by what they mean?

Line 835: the authors indicate a seeming disconnect between the vast transcriptome change and the lack of changes in protein abundance, it is likely reflected that the average mitochondrial protein half-life is ~ 20 days.

Line 912: to some extent, the use of a bed-rest protocol to understand inactivity seems inappropriate. This relatively acute study on bed-rest seems most analogous, as the authors pointed out, to periods of hospitalization. Arguably, even inactivity/step reduction studies are not as deleterious to health as bedrest studies.

END OF COMMENTS

EDITOR COMMENTS

Reviewing Editor:

Thank you for submitting your work to The Journal of Physiology. Two reviewers have provided a thorough review and although they were enthusiastic about the merits of the work, both sought additional clarification and revision. With respect to reviewer 2's questions about the use of CS activity to normalize the respirometry rather than EM-determined mitochondrial volume density, I would suggest that you specify in the Discussion the rationale for this was that there could be disparity between the EM-based mitochondrial volume density and the CS activity in the muscle bundle since the measures were performed on separate pieces of muscle from the same biopsy. You should also consider addressing the potential limitations of using CS activity as an index of mitochondrial content in this same section how this might impact the interpretation of elevated mitochondrial respiration per unit of mitochondrial volume.

We thank the Reviewing Editor for this important comment. We agree that citrate synthase (CS) activity represents an indirect proxy of mitochondrial content and that electron microscopy-derived mitochondrial volume density provides a more direct structural assessment. We also acknowledge that these measurements were obtained from different muscle bundles, which may contribute to differences in interpretation. And the strong point for using CS for normalisation is in the fact that it allows the assessment of the mitochondrial content in the same biopsy piece as the one where the respirometry was done.

In response, we have revised the Discussion to clarify the rationale for the use of CS activity in normalising respirometry data and to explicitly acknowledge its limitations as an index of mitochondrial content. We have also clarified how this may influence the interpretation of respiration normalised to CS activity. Lines 60,62-63,500-503, 662-664,811-829,990-994.

Senior Editor:

Thank you for submitting your manuscript for consideration by The Journal of Physiology. As part of the peer review process, we recruited two Referees with expertise in this field of study. Both referees noted that there is potential for your manuscript to be influential in this field of study, however they also both noted some concerns that would need to be satisfactorily addressed for this impact to be realized. At this point we would like to invite you to respond point-by-point to each Referee's concerns, making corresponding revisions to your manuscript. We look forward to seeing your revised work and appreciate your interest in The Journal of Physiology!

Thank you for your attention to The Journal's statistical reporting policy. Could you please revisit Figure 11A to provide precise p-values as you have with the other figures? Thank you.

We thank the Senior Editor for this important comment. We have revised Figure 11 to include exact p-values for all reported comparisons in the figure caption.

Referee #1:

Dear Motanova et al.,

I have evaluated your manuscript and find it clear, well written, and potentially important. Below are my comments for your consideration.

We thank the Reviewer for the positive evaluation of our manuscript and for the constructive comments. Below, we provide a point-by-point response to each comment, indicating the corresponding changes made in the revised manuscript. The responses to each comment are highlighted in purple, while the corresponding changes introduced in the revised manuscript are highlighted in grey.

Major comments

1. I remain skeptical about the use of phrases such as "the combined effects of ageing and disuse" and "their combined effects on...". To me, this implies an interaction between ageing and disuse. However, the study assess the effects of disuse in older adults, not an ageing × disuse interaction. This distinction is important and should be made clear throughout the manuscript.

We thank the Reviewer for pointing this out and fully agree with this distinction. Our study investigates the effects of disuse within an older population, not ageing and disuse interaction. Accordingly, we have refined the wording throughout the manuscript to clarify this point (lines 52-54,86-89,142-143).

2. It is my understanding that the project on which this manuscript is based has also been used for other publications. This is of course acceptable, but should be handled transparently. Please clarify whether data from the same participants have been published previously, and if so, where.

We confirm that no previous publications have reported results on the same research topic using data from the participants included in the present study. Therefore, the findings presented in this manuscript are original and have not been published elsewhere.

However, some participants involved in this project have contributed to other publications addressing different research questions and topics. To ensure transparency, we report below the corresponding DOIs of the related publications: 10.1113/JP288448; 10.1113/EP092709.

3. Related to this, was muscle atrophy actually induced by the bed-rest model? In other words, did the model work as intended? This should either be shown in the present manuscript or clearly referenced if already reported elsewhere.

We agree that confirming the efficacy of the bed-rest model is crucial for the context of this study. The 10-day bed-rest protocol successfully induced significant muscle atrophy, architectural remodelling, and strength loss. These changes were tracked and confirmed by our collaborative research group via magnetic resonance imaging (MRI), ultrasound, and dynamometry. Because these data belong to a broader, interconnected project currently in preparation for standalone publication, we cannot present the full raw data sets within this manuscript. However, the foundational validation metrics have been published in the official book of abstracts (Scarpelli, M. C., Colosio, M., Simunič, B., Pisot, R., Alcazar, J., Bonato, M., Porcelli, S., Narici, M., & Franchi, M. V. (2025). Endurance training as an effective strategy to counteract impairments in muscle mass and function after 10 days of bed rest in older adults. *Physiology*, *40*(S1), 2122. https://doi.org/10.1152/physiol.2025.40.S1.2122).

In accordance with the Reviewer's suggestion, we have updated the manuscript (Lines 481-485) to explicitly summarise these validation metrics. Briefly, 10 days of bed rest resulted in significant structural and functional declines, including decreases in quadriceps femoris volume (-6.2%, $p < 0.001$) and muscle thickness (-7.4%, $p = 0.02$). Functional impairment was confirmed by a -13.3% decrease in maximum voluntary isometric contraction ($p < 0.001$).

4. If BR0 and BR10 RNA-seq samples are paired within individuals, the differential expression model should account for participant identity. A model including only Time treats observations as independent and may fail to account for substantial inter-individual baseline variation.

We thank the reviewer for this important methodological point. In the revised analysis, we explicitly modelled the paired design of the RNA-seq data by including participant identity in the DESeq2 design formula. Specifically, we used only the coding-sequence (CDS) count matrix and fitted a model of the form design = ~ Subject + TimePoint, where "Subject" denotes the individual participant and "TimePoint" has two levels (PRE, BR10). Differential

expression was then assessed using the BR10 vs PRE contrast, which estimates the effect of bed rest within individuals while accounting for inter-individual baseline variability.

Genes with fewer than 10 total counts across all samples were removed prior to modelling, counts were normalised and dispersions estimated using DESeq2's internal procedures, and P-values were adjusted using the Benjamini–Hochberg method. Genes with adjusted P-value (padj) < 0.05 were considered significantly differentially expressed, without applying any $\log_2$ fold-change threshold in the statistical test. Thresholds on $|\log_2 FC|$ were used only for descriptive classification and plotting (e.g. volcano plots). The main conclusions of the study were unchanged after adopting the paired model.

We have revised the Methods section accordingly to describe the paired design.

To visualise the within-subject structure of the data under the revised model, we also generated a paired PCA plot based on variance-stabilised CDS counts (new Fig. 9.1). In this representation, PRE and BR10 samples from the same participant are connected by a line. The plot illustrates both clear separation between PRE and BR10 along the first principal component (PC1: 32% of the variance) and coherent within-subject shifts, supporting the robustness of the paired design and the absence of outlying samples.

5. Citrate synthase activity and EM data suggest lower mitochondrial content, whereas Complex I-V protein abundance normalized to β-actin appears unchanged. Please discuss this apparent discrepancy. Does this indicate preserved OXPHOS protein abundance despite lower mitochondrial content, altered mitochondrial composition, or possible limitations in the sensitivity of the protein measurements?

The reduction in citrate synthase activity and mitochondrial volume density observed by TEM suggests a decrease in mitochondrial content following inactivity. In contrast, the abundance of OXPHOS Complex I–V proteins normalised to β-actin was not significantly altered.

Several factors may explain this apparent discrepancy. First, the magnitude of the observed reductions in mitochondrial content was relatively modest (1.48-fold for citrate synthase activity and 1.27-fold for mitochondrial volume density). Such changes may fall below the detection threshold of Western blot-based quantification, particularly in human skeletal muscle, which is characterised by higher biological variability than cell or animal models.

Second, citrate synthase activity, mitochondrial volume density, and OXPHOS protein abundance reflect distinct aspects of mitochondrial organisation and are not necessarily expected to change in a strictly proportional manner.

Third, previous bed rest studies in older individuals (Standley et al., 2020. doi:10.1093/gerona/glaa001) have reported significant reductions in OXPHOS complex abundance following 10 days of inactivity, supporting the notion that respiratory chain proteins are responsive to disuse, although the magnitude of change is context dependent.

Finally, transcriptional downregulation of OXPHOS-related genes in our dataset suggests early molecular remodelling of mitochondrial pathways, which may precede detectable alterations at the protein level. In addition, differences in protein turnover kinetics may also contribute, as mitochondrial respiratory chain proteins generally exhibit relatively slow turnover, potentially leading to a temporal delay between transcriptional changes and measurable protein-level adaptations during short-term interventions.

We have now included a paragraph in the Discussion to address this point (Line 830-845)

6. For Table 7/Figure 7, please clarify whether p-values were adjusted for multiple comparisons across the mitochondrial protein panel. If not, please apply an appropriate correction or clearly state that these analyses are exploratory.

We thank the Reviewer for this important recommendation. Following this suggestion, we have applied a Bonferroni correction to account for the nine comparisons within the mitochondrial fission and fusion protein panel. Consequently, the threshold for statistical significance was set at $p \leq 0.0056$ ($\alpha = 0.05 / 9$). Following this adjustment, the change in MFN1 protein abundance is no longer statistically significant. We have updated the statistical data in Figure 7 and adjusted the text and figure legends throughout the manuscript to reflect this outcome accurately.

7. I find the correlation analysis between mitochondrial fusion/fission proteins and ROS production problematic as currently presented. The more correlations that are tested, the greater the chance of finding a significant one. If the strongest correlation as shown is borderline significant and no correction for multiple testing has been applied, the evidence for a robust link is weak. If you choose to keep this analysis, please state explicitly that it is exploratory and report exactly how many correlations were tested. A summary table of all correlations may also be useful.

We thank the reviewer for this comment. The point is well taken regarding the risk of type I error associated with multiple correlation testing and the exploratory nature of these analyses.

In light of this concern, we have decided to remove the correlation analyses between mitochondrial fusion/fission proteins and ROS production from the revised manuscript. These analyses were originally performed in an exploratory context; however, we agree that, in their current form, they may be misleading without appropriate correction for multiple testing and a more comprehensive presentation of all tested associations.

8. Please clarify whether the GO-CC analysis, which was mentioned in methods, provided any information related to mitochondrial number, composition, or mitochondrial compartments.
GO-CC enrichment was used to identify subcellular compartments that are over-represented among differentially expressed genes, rather than to estimate mitochondrial number directly. In the revised analysis, GO-CC analysis highlighted several

mitochondrial-related components, including the mitochondrial inner membrane, mitochondrial matrix, and respiratory chain complexes, consistent with the broad suppression of mitochondrial respiratory and translational machinery observed at the transcript level. We have now included these results in Fig. 10 to provide a more comprehensive view of the affected mitochondrial compartments.

However, GO-CC enrichment describes the distribution of regulated genes across annotated cellular components (i.e. which compartments are enriched in DEGs), and does not provide a quantitative measure of organelle abundance per se. For this reason, we relied on citrate synthase activity and electron microscopy to assess mitochondrial content and morphology.

Specific comments

• Is there a particular reason why p-values and effect sizes are inserted in tables above each figure? These could perhaps be shown directly in the corresponding figure, where relevant.

We appreciate the Reviewer’s valuable feedback regarding the presentation of our data. Initially, we chose to omit the exact p-values and effect sizes from the figures to avoid cluttering the panels, particularly given the large number of individual graphs included in some of the figures. However, we completely agree that displaying this information directly on the graphs significantly enhances readability and simplifies data interpretation for the reader. Following the Reviewer’s suggestion, we have updated the figures to include both the precise p-values and effect sizes directly on the panels and adjusted the figure legends.

Furthermore, to maximise methodological transparency, we have expanded the Statistical Analysis section in the Methods to include the explicit criteria used for evaluating our effect sizes, which was previously missing.

• In general, mitochondrial biology papers contain many abbreviations. I recommend defining the relevant abbreviations in each figure legend to improve readability.

We thank the Reviewer for this suggestion. We agree that defining abbreviations directly within the legends significantly enhances the readability and accessibility of the figures. We have systematically revised every figure legend throughout the manuscript to ensure that all relevant abbreviations are explicitly defined.

• Figure 2 and others: The subfigure numbering should be consistent throughout the manuscript. Please use one system. Refine the numbering

We appreciate the Reviewer pointing this out. We have thoroughly reviewed and refined the subfigure numbering system across all figures in the manuscript to ensure absolute consistency. All panels are now standardised using a single uniform numerical system (e.g. 1, 2, 3) throughout both the figures and their corresponding legends.

• Figure 6A: Please examine the clear outlier. The value appears approximately 7-8-fold higher than all others, raising the possibility of a methodological issue. If retained, please justify its inclusion; alternatively, consider presenting analysis with and without this value.

We thank the reviewer for this important observation. We performed a formal outlier assessment using Grubbs' test, and the value in question was identified as a statistical outlier. We carefully reviewed the original data and experimental records and found no evidence of methodological, technical, or data processing issues. Nevertheless, to avoid any potential bias, the outlier was excluded from the final analysis.

To clarify this point, we have revised the figure legend to indicate that the analysis was performed on n = 8 participants, as one participant was identified as a statistical outlier and excluded from the analysis.

• Line 106-107: "As the primary site of energy production within muscle cells..." This is too broad. Mitochondria are the primary site of oxidative ATP production, but not all energy production.

We thank the Reviewer for this important clarification. We agree that the original wording was too broad. We have revised the sentence to specify that mitochondria are the primary site of oxidative phosphorylation-mediated ATP production, rather than all cellular energy production. The revised sentence now reads: “As the primary site of oxidative phosphorylation-mediated ATP production within muscle cells, mitochondria support both contractile activity and the metabolic processes essential for muscle maintenance.”

• Line 155-156: "Ten older males (mean age: 68.5 {plus minus} 2.64 years; height: 172.7 {plus minus} 6.31 cm; body mass: 85.6 {plus minus} 12.25 kg..." Please adjust decimals to a level that reflects realistic measurement precision.

We thank the reviewer for this important comment. We agree that the originally reported values included excessive decimal precision relative to the measurement accuracy of anthropometric variables. The updated sentence now reads: “Ten older males (mean age: 68.5 ± 2.6 years; height: 172.7 ± 6.3 cm; body mass: 85.6 ± 12.3 kg).”

• Line 175: "the ambulatory period" is unclear in this context. Please define.

We thank the Reviewer for allowing us to clarify this point. The "ambulatory period" refers to the baseline measurement period, during which participants came to the hospital 2 days before the bed rest onset to familiarise with the procedures and perform the initial *in vivo* assessments. We adjusted this information in the text on Lines 192-193.

• Line 180-181: "...from contralateral legs before (PRE) and after (BR10) the bed rest intervention." It is not clear what "contralateral" refers to here.

By “contralateral” leg we meant that biopsies were taken from the opposite leg at each time point to avoid repeated sampling from the same site: from the right leg at PRE and the left leg at BR10. We have clarified this point better in the manuscript on Line 199.

• Line 508-511: The reported p-values reported in text do not appear to correspond to the values shown in Table 3. Please clarify which comparisons these p-values refer to. Change based on table

We thank the Reviewer for bringing this issue to our attention. An inconsistency was present between the p-values reported in the text and those presented in Table 3. The values reported in Table 3 were correct, and the corresponding section of the manuscript has been revised to ensure full consistency between the text and the table (see line 543 and Fig.3.1). In addition, following the Reviewer’s previous suggestion, the precise p-values have now been indicated directly on the figures, and the tables have been removed from the manuscript.

• Line 644-646: The total number of counted mitochondria is less informative than the number of mitochondria analysed per subject/sample. Please provide the latter.

We thank the Reviewer for this valuable suggestion. We agree that reporting the number of mitochondria analysed per subject is more informative. In the revised manuscript, we have therefore included the mean number of mitochondria analysed per subject for each mitochondrial subpopulation (IMF and SS) and time point (PRE and BR10), in addition to the total number of mitochondria traced for transparency. This information is now reported in the Results section. Lines 673–676.

• Line 695: Please define what is meant by "strongly downregulated", "strongly upregulated", and "moderately regulated" in this context.

We apologise for the lack of clarity. In the revised manuscript we explicitly define these terms based on the shrinkage-adjusted $\log_2$ fold-changes obtained from the DESeq2 model (BR10 vs PRE) and the adjusted P-value threshold. Specifically, among genes with padj < 0.05:

“Strongly upregulated” genes are those with $\log_2FC \geq 1$
“Strongly downregulated” genes have $\log_2FC \leq -1$
“Moderately regulated” genes have $0.5 \leq |\log_2FC| < 1$.

These categories are used only for descriptive purposes in Figure 9.2. The statistical inference is based solely on padj < 0.05 without imposing a $\log_2FC$ cut-off in the model itself. We have updated the Results section and panel 2 in Figure 9 to include these explicit definitions when reporting the numbers of regulated genes.

Referee #2:

Motanova and colleagues present a thorough investigation into the effects of 10 days of bed rest in older adults on mitochondrial respiration, volume, and morphology. The authors report that while 10-days of bed rest had no effect on respiratory capacity, there was an increase in ROS emission. The authors also share data that suggest that mitochondrial protein complex abundance was unchanged as was supercomplex formation. The authors suggest that modulation in ROS emission may have been facilitated by increased mitochondrial fission as measured by markers of mitochondrial dynamics and a decrease in mitochondria (presumably by increased mitophagy) as measured by TEM. Finally, the others present transcriptomic data that suggests that 10 days of bed rest led to mitochondrial remodeling, increasing expression of genes related to redox and mitophagic pathways. Overall, the study presented is very well-done and the findings are interesting. A similar bed-rest model has been employed in adults prior by a different group. These data seem to conflict with some of their findings (present study does not report a decline in respiratory capacity, the prior study did). It would seem appropriate to acknowledge this study to greater extent. Otherwise, there are no major concerns, only some suggestions in hopes to improve the manuscript. This is an otherwise well-written and well-presented study.

We are very grateful to the Reviewer for taking the time to evaluate our manuscript and for their highly encouraging and appreciative comments. Below, we provide a point-by-point response to each comment, indicating the corresponding changes made in the revised manuscript. The responses to each comment are highlighted in purple, while the corresponding changes introduced in the revised manuscript are highlighted in grey.

We completely agree with the Reviewer’s suggestion that a more comprehensive comparison with the work of Standley et al. (2020) (10.1093/gerona/glaa001), in which the authors also utilised a 10-day bed rest model in older adults, would greatly enrich our discussion. As the Reviewer noted, Standley et al. (2020) reported a decline in respiratory capacity alongside a reduction in OXPHOS complex protein abundance, whereas our cohort demonstrated preserved respiratory efficiency and unchanged OXPHOS protein levels. In the revised manuscript, we have expanded our discussion to explicitly address these conflicting observations. Line 830-845.

1) It is odd the authors rely on citrate synthase activity as a metric for mitochondrial mass when the authors have gone through the very rigorous process of measuring mitochondrial density. Previous papers have demonstrated that citrate synthase might be moderately correlated with citrate synthase but is poor at predicting changes in mitochondrial volume (28580772). Thus, using citrate synthase activity as the indicator that mass changed is odd (example: line 754)
For that reason, the authors ought to consider expressing mitochondrial respiration relative to muscle mass and mitochondria mass/density instead of citrate synthase activity. Additionally, out of the sake of rigor and continuing to evaluate the utility of citrate synthase as a marker of mitochondrial mass/density, would the authors consider checking if:

1. Citrate synthase activity correlates with measures of mitochondrial density?
2. Changes in CS activity predicts changes in changes in mitochondrial density?

We thank the reviewer for this insightful comment, which addresses an important discussion in the field regarding the use of CS activity as a proxy for mitochondrial content.

We fully agree that CS activity is an indirect biochemical marker and does not represent a direct measurement of mitochondrial mass. As previously reported in the literature (e.g., 10.1111/apha.12905), the relationship between CS activity and mitochondrial volume density assessed by electron microscopy is only moderate, and CS should therefore be interpreted as an indirect indicator of mitochondrial content rather than a direct measure of mitochondrial mass.

In the present study, mitochondrial volume density was directly quantified by electron microscopy, providing a structural assessment of mitochondrial abundance. This represents a major strength of our work and allows for a more accurate interpretation of changes in mitochondrial content independently of enzymatic proxies.

The rationale for also reporting CS activity lies in its widespread use in the literature as a biochemical surrogate of mitochondrial content and, importantly, in the methodological advantage that both CS activity and mitochondrial respiration were assessed in the same bundles of permeabilised muscle fibres. This approach enables direct comparison of functional and enzymatic measurements within the same biological sample and facilitates interpretation in the context of existing literature.

Following the Reviewer's suggestion, to evaluate the relationship between citrate synthase (CS) activity and mitochondrial structural content, we first examined the correlation between CS activity and mitochondrial volume density using fold-change values relative to baseline. This analysis revealed a weak association (Pearson's $r = 0.19$, $p = 0.65$), indicating no significant relationship between baseline-normalised CS activity and mitochondrial volume density. We additionally performed a linear regression analysis assessing the relationship between changes (Δ) in CS activity and mitochondrial volume density. This analysis demonstrated a weak-to-moderate positive association ($r = 0.54$, $R^2 = 0.29$), which

did not reach statistical significance (p = 0.17). Together, these findings indicate that neither CS activity nor changes in CS activity were significantly associated with mitochondrial volume density in our cohort. Accordingly, we have revised the manuscript to clarify that CS activity should be interpreted as an enzymatic marker of mitochondrial content and function, rather than as a direct surrogate measure of mitochondrial mass. Lines 60,62-63,500-503, 662-664,811-829,990-994.

2)Members of the study team have previous published a technique of investigating mitochondrial ADP sensitivity using a two-phase approach in the young bed-rest cohort (40285367). Why did the authors not employ that approach on these data?

We thank the reviewer for this comment. We would like to clarify that the two-phase approach for assessing mitochondrial ADP sensitivity was already implemented in the present study, as described in the Methods section (Lines 266-270) and based on our previously published methodology (Zuccarelli, De Martino et al., 2025; 10.1002/jcsm.13775).

In the revised manuscript, we have further clarified that the apparent Km reported in the study was calculated as the ADP concentration corresponding to 50% of maximal respiration. This information has now been explicitly stated in the Methods section (Lines 264-269).

For the reviewer's convenience, we also report here the values obtained for the first and second phases of the analysis (Km1 and Km2, as well as Vmax1 and Vmax2). Two-way ANOVA revealed no significant effect of bed rest (PRE vs. POST) on Km ($p = 0.229$) or Vmax ($p = 0.234$). In contrast, a significant effect of phase was observed, with Km1 differing from Km2 ($p = 0.001$) and Vmax1 differing from Vmax2 ($p < 0.001$), confirming the expected differences between the two phases of the ADP titration protocol.

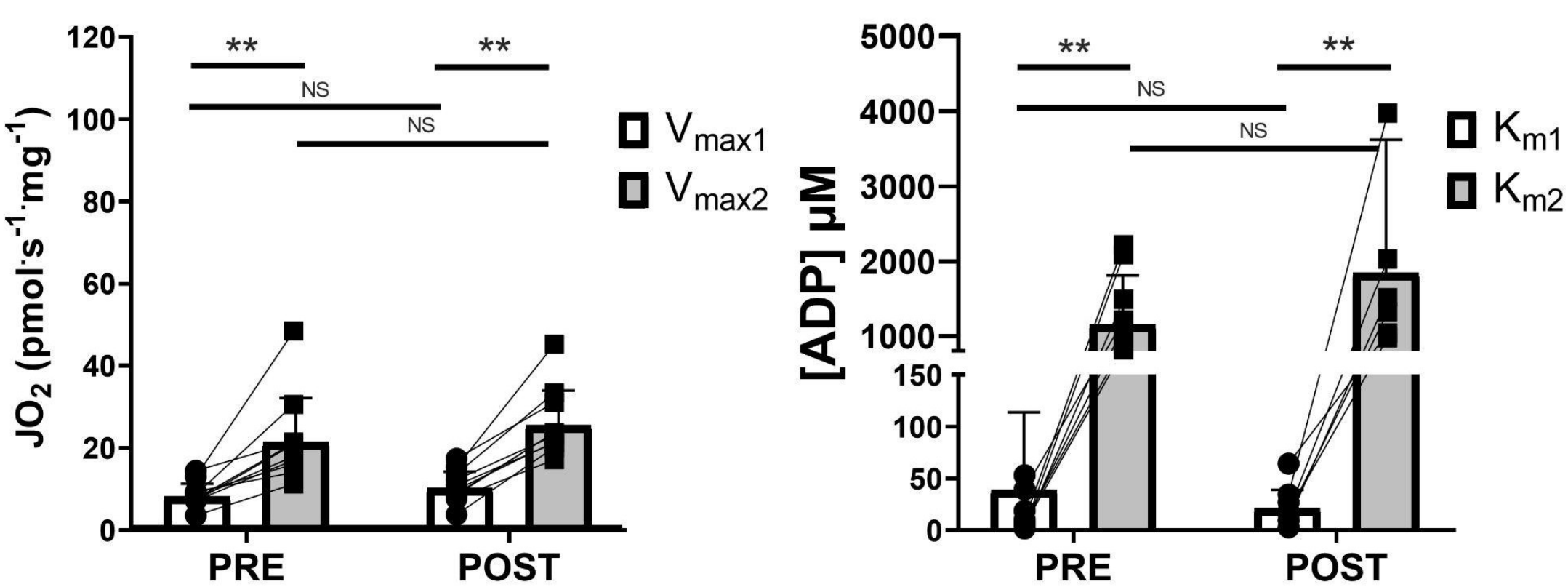

3) The interrogation of mitochondrial fission and fusion may be strengthened if the authors published more TEM data. Is it possible to report average surface area/volume of mitochondria pre and post bed rest? Presumably, increased fission and decreased fusion would drive for smaller sized mitochondria lending to a more fragmented phenotype.

We thank the reviewer for this insightful comment. We agree that an imbalance in mitochondrial fission and fusion processes could, in principle, lead to a fragmented mitochondrial phenotype characterised by smaller and more spherical organelles.

To address this point, we clarify that our transmission electron microscopy (TEM)-based morphometric analysis was specifically designed to detect such structural alterations using established quantitative shape descriptors. As described in the Methods, mitochondria were manually traced to calculate aspect ratio (AR), defined as the ratio of the major to minor axis and reflecting mitochondrial elongation, and form factor (FF), calculated as $perimeter^2/4\pi*area$ and reflecting mitochondrial branching complexity. These parameters are widely used in mitochondrial morphometry to describe organelle shape independently of size (Picard et al., 2013. 10.1152/japplphysiol.01096.2012).

In both intermyofibrillar (IMF) and subsarcolemmal (SS) mitochondrial populations, neither AR nor FF was significantly altered following 10 days of bed rest. This indicates that mitochondrial shape characteristics, including elongation and branching complexity, were preserved despite the intervention. Accordingly, these data do not support the presence of an overt shift toward a fragmented mitochondrial phenotype at this time point.

To fully address the reviewer's request, we provide the graphs for mitochondrial area and perimeter below. Furthermore, we have expanded the corresponding section of the Discussion to more explicitly articulate these architectural findings and their biological implications. Lines 913-919.

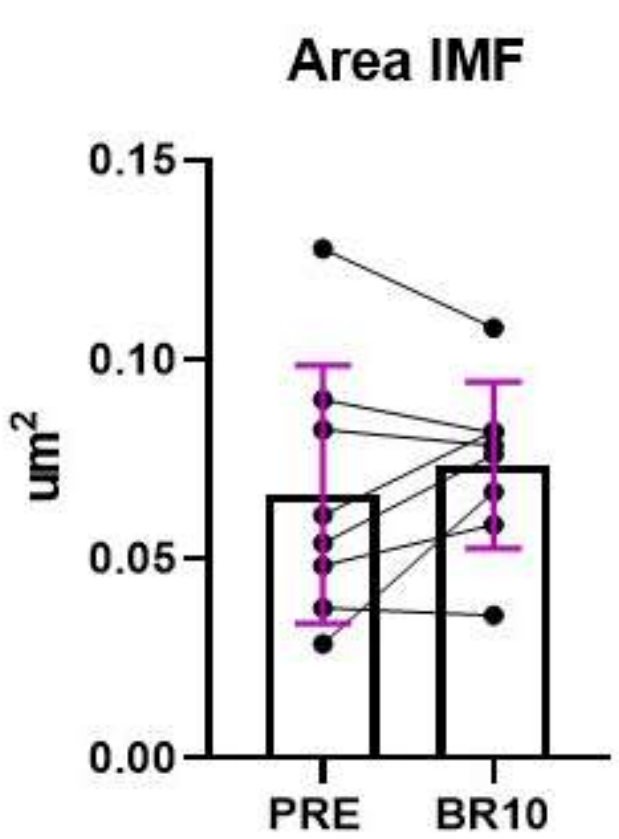

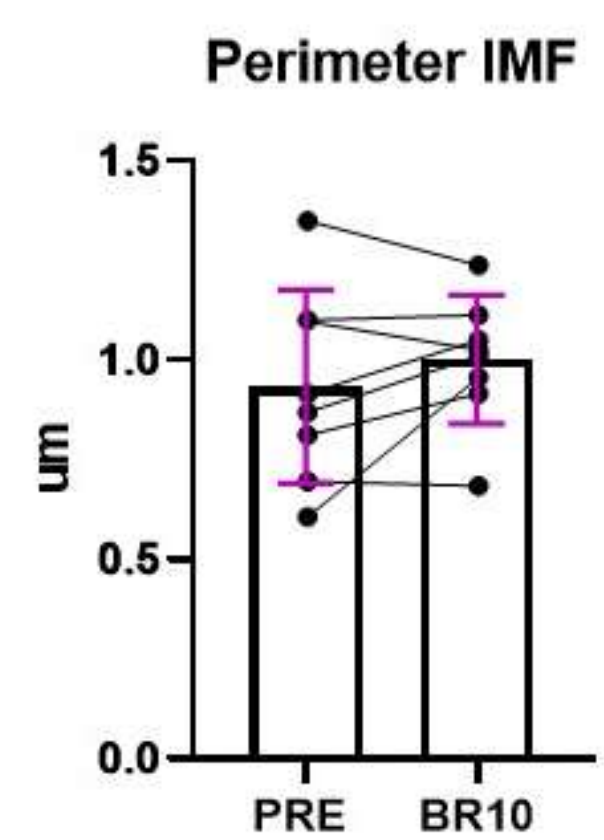

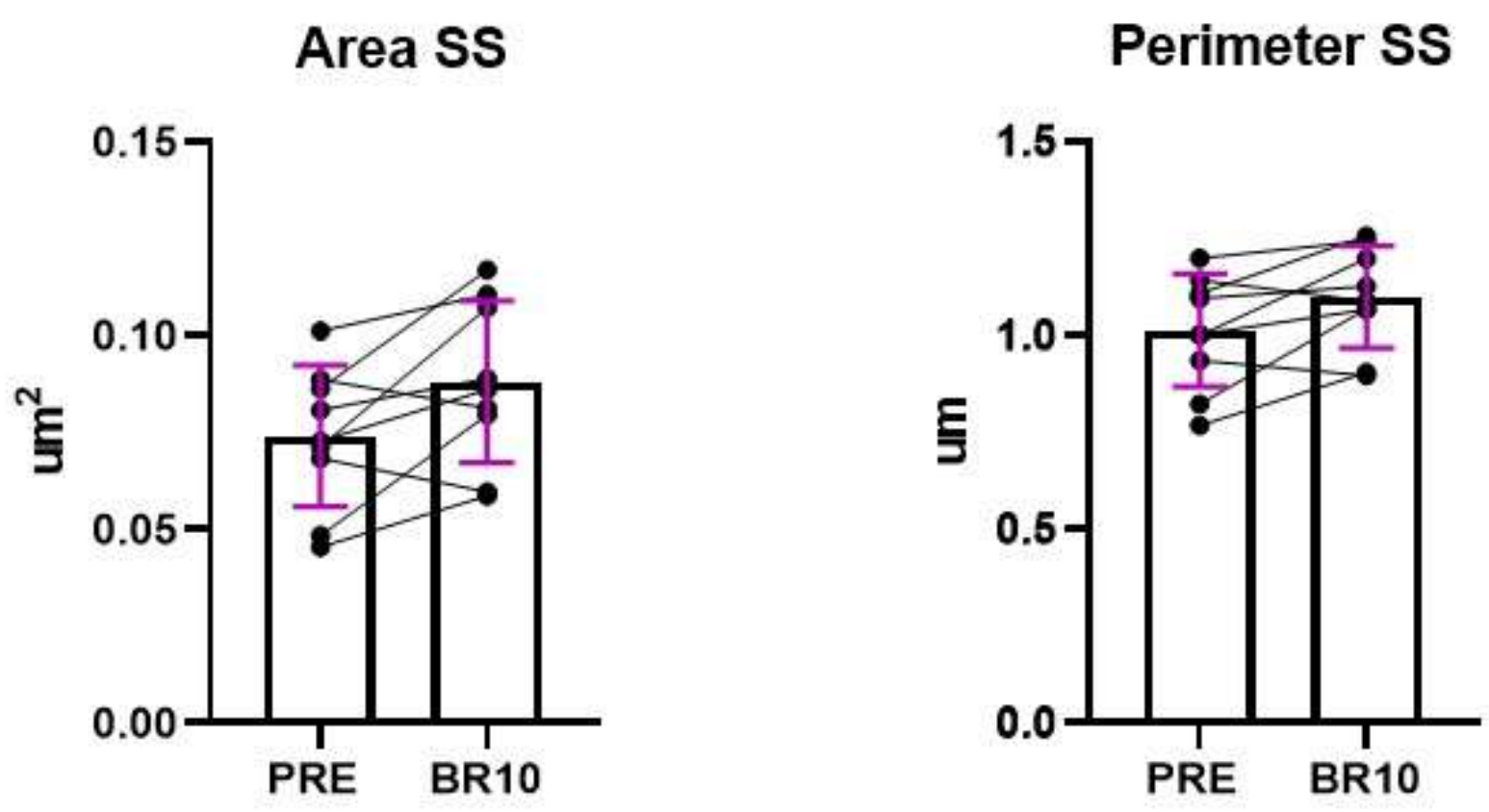

Regarding the reviewer's specific interest in mitochondrial volume, we would like to highlight that the overall mitochondrial volume density data are already fully evaluated and presented in this manuscript. The reviewer can find these detailed volumetric results reported on Lines 665-669 and visually represented in Figure 8 (panels 7-9) of the main text.

4) Line 764: "...no changes were detected in canonical mitophagy proteins... indicating that contribution of mitophagy cannot be definitely established..." did the authors probe for protein expression related to mitochondrial biogenesis?

We appreciate the opportunity to clarify this point. In our original western blot panel we focused on proteins related to mitochondrial dynamics and mitophagy, and did not include canonical markers of mitochondrial biogenesis such as PGC-1α or TFAM. We therefore cannot directly assess mitochondrial biogenesis at the protein level in this dataset. Additional western blot analyses of these markers could not be performed, as the available muscle lysates had been fully consumed during the original experimental analyses.

To address the reviewer's concern at the transcriptomic level, we extended our pathway analysis to include a curated mitochondrial biogenesis gene set corresponding to GO:0007005 ("mitochondrion organisation"), from which we extracted a biogenesis-focused subset of genes (*PPARGC1A, NRF1, NFE2L2/NRF2, TFAM, TFB1M, TFB2M, TOMM40, TIMM23*) in addition to the broader mitochondrial organisation components. Using Fisher's exact test, we observed significant over-representation of differentially expressed genes (padj < 0.05) within this mitochondrial biogenesis set (odds ratio ≈ 3.7, adjusted P ≈ 3×10^{-25}), indicating a robust enrichment at the pathway level (Fig. 11.1).

However, inspection of individual genes within this panel revealed that *PPARGC1A* and *TFAM* themselves showed only modest, non-significant downward trends, whereas *NRF1* was significantly downregulated and *NFE2L2* (encoding *NRF2*) was significantly upregulated. Several downstream genes related to mitochondrial organisation, protein import and mitochondrial gene expression also contributed to the enrichment signal. Overall, these data suggest that short-term bed rest in older adults is associated with coordinated

remodelling of biogenesis-related pathways and mitochondrial organisation, rather than a simple, large-amplitude induction of classical biogenesis master regulators at the transcript level.

5) It is interesting that despite a decrease in mitochondrial volume, there was no change in mitochondrial OXPHOS complex protein expression. To one extent, this may suggest that only certainly mitochondrial proteins (unrelated to OXPHOS) are targeted for degradation and/or the matrix shrinks in size.

We thank the Reviewer for this insightful comment. We agree that the observed dissociation between reduced mitochondrial volume density and preserved OXPHOS complex protein abundance may reflect heterogeneous remodelling of mitochondrial structure and composition during disuse. One explanation is that reductions in mitochondrial content primarily affect organelle volume, while OXPHOS protein abundance within the remaining mitochondrial network is relatively preserved over this time frame. In addition, the relatively modest magnitude of the reduction in mitochondrial volume density may fall below the detection sensitivity of semi-quantitative Western blot analysis in human skeletal muscle, which is subject to higher biological variability. Finally, differences in the temporal dynamics and turnover rates of mitochondrial structural components versus respiratory chain proteins may contribute to a delayed or non-proportional relationship between changes in mitochondrial volume and OXPHOS protein abundance. We have altered the corresponding paragraph in the Discussion to address this point. Lines 830-845.

6) Line 875: the authors suggest that respiration was enhanced on a "per-mitochondrion" basis. However, the authors did not count the number of mitochondria. Rather, they provide mitochondrial mass/volume.

We agree that the term “per-mitochondrion basis” was not appropriate, as mitochondrial number was not directly assessed in the present study.

To address this point, we have revised the manuscript to avoid any implication regarding mitochondrial number. The revised text now clearly states that the observed increase reflects enhanced intrinsic mitochondrial function relative to mitochondrial content rather than changes on a per-mitochondrion basis (see lines 990-994).

7) Line 890: the authors indicate that central factors may determine decline in oxidative capacity. Are they referring to oxidative capacity at the mitochondrial, muscle, or human-level? On that note, did the authors evaluate any physical outcomes (VO2max, DXA, muscle volume, strength)?

We thank the Reviewer for this comment and agree that our original wording was not sufficiently specific, which may have led to ambiguity regarding the level at which “oxidative capacity” was intended.

Our intention was to indicate oxidative capacity at the systemic level, as classically reflected by maximal oxygen uptake (VO_2max), rather than mitochondrial or isolated muscle oxidative function. We have therefore clarified this point in the revised manuscript to avoid any possible misinterpretation.

We also appreciate the Reviewer's question regarding functional outcomes. While these data were not included in the present manuscript, we have previously evaluated related physiological and functional parameters in the same experimental study. In particular, as presented at the American College of Sports Medicine Annual Meeting (2025, Doi: 10.1249/01.mss.0001156072.84865.d8; conference abstract), we observed a reduction in VO_2max (both in absolute terms and normalised to body mass) following bed rest, together with impairments in microvascular function. These findings are currently in preparation for full publication.

Accordingly, we now explicitly indicate that the discussion refers to oxidative metabolism at the whole-body level during maximal exercise (i.e., VO_2max), which represents the integrated physiological outcome of cardiovascular, microvascular, and muscular oxygen transport and utilization. The original text has been modified accordingly (see lines 1002-1005).

In addition, the bed-rest intervention induced significant structural and functional deterioration of skeletal muscle, assessed using magnetic resonance imaging (MRI), ultrasound, and dynamometry. These analyses demonstrated significant reductions in quadriceps femoris volume (-6.2%, $p < 0.001$), muscle thickness (-7.4%, $p = 0.02$), and maximal voluntary isometric contraction (-13.3%, $p < 0.001$). These outcomes are part of a broader, interconnected project that is currently being prepared for standalone publication, however, summary-level data have already been reported in an official conference abstract (https://doi.org/10.1152/physiol.2025.40.S1.2122). In line with the Reviewer's suggestion, we have therefore included a summary of these validation metrics in the revised manuscript (Lines 481-485).

8) This was a resource intensive study. Understandably, it is unlikely that there will be more data coming from this study. However, it would be very interesting to understand how mitochondria recover after bed-rest is discontinued and normal activity is resumed. Understanding the recovery (or lack thereof) seems just as important to understand.

We agree with the Reviewer's suggestion regarding the importance of studying recovery periods. In the present experimental design, following the bed rest period, participants were required, for ethical reasons, to complete a 3-week aerobic retraining program to facilitate the restoration of baseline fitness levels.

The study referenced in our previous response that is, as mentioned previously, currently under preparation as a standalone publication, will specifically focus on the recovery phase

following inactivity. Given that endurance training is well known to enhance oxidative metabolism, this follow-up study aims to investigate whether such an intervention can also reverse disuse-induced impairments in skeletal muscle. Importantly, this complementary study will not include the mitochondrial functional and structural parameters assessed in the present manuscript, except for transcriptomic analysis.

We also agree that future studies could benefit from more controlled experimental models of reduced physical activity (e.g., hindlimb suspension or limb casting in animal models), allowing direct comparison between active recovery, continued inactivity, and normal activity conditions. Such approaches would enable a more detailed characterisation of mitochondrial adaptations across different recovery and disuse scenarios. We have added a paragraph discussing these perspectives to the discussion section. Line 1026-1039.

Minor comments

Line 387: it is a significant undertaking to manually trace mitochondria. Was this done by one individual? If done by more than 1 individual, was there any internal verification/inter-rater tests done to ensure no bias? Were these individuals blinded to samples? Were the number of mitochondria identified similar from participant to participant?

Two independent, blinded operators performed all mitochondrial analyses, including assessments of mitochondrial volume density and morphology, without knowledge of participant identity or study timepoint. To evaluate operator-related variability and minimise the risk of bias, we calculated the inter-operator coefficient of variation (CV). The inter-operator CVs were 4.5% for mitochondrial area, 3.3% for perimeter, 3.6% for aspect ratio, 2.0% for form factor, and 5.9% for mitochondrial volume density.

During mitochondrial morphology analyses, all mitochondria within the selected images were quantified. The total number of mitochondria identified by the two operators showed an inter-operator CV of 5.7%.

We have currently included these data to the methods section on Lines 413-419.

Figure 8 and Table 8: will the authors consider not using abbreviations for the graphs and if possible, also for the table?

We thank the Reviewer for this suggestion. We have ensured that all abbreviations are fully defined in the figure legends to improve clarity and readability. In addition, where applicable, abbreviations have been refined to enhance consistency across figures and tables.

Line 803: the authors reference "subtle remodeling of cellular redox regulation" could they be more explicit by what they mean?

We thank the Reviewer for this comment. We have refined the wording in this section to better reflect the observed transcriptional changes. Lines 768-788.

Line 835: the authors indicate a seeming disconnect between the vast transcriptome change and the lack of changes in protein abundance, it is likely reflected that the average mitochondrial protein half-life is ~ 20 days.

We thank the Reviewer for this suggestion regarding mitochondrial protein kinetics. In the original manuscript, this point was addressed briefly and in a more speculative manner. In response, we have expanded this section of the Discussion (Lines 947-957) to provide a more balanced interpretation of the observed transcript–protein discordance. Specifically, we now include evidence from in vivo proteomic studies indicating that some mitochondrial proteins have relatively long half-lives on the order of weeks, which may explain the absence of marked changes in protein abundance following 10 days of bed rest. We also contrast this with established literature reporting faster turnover rates for selected mitochondrial proteins, thereby placing our findings within a temporal framework of mitochondrial protein dynamics during early disuse.

Line 912: to some extent, the use of a bed-rest protocol to understand inactivity seems inappropriate. This relatively acute study on bed-rest seems most analogous, as the authors pointed out, to periods of hospitalization. Arguably, even inactivity/step reduction studies are not as deleterious to health as bedrest studies.

We thank the Reviewer for this insightful comment. We agree that bed rest represents an extreme and highly controlled model of physical inactivity, which does not aim to reproduce habitual reductions in daily activity. Instead, its primary strength lies in modelling acute, complete physical inactivity under tightly controlled conditions.

In the present study, our intention was to use bed rest as an experimental analogue of clinically relevant situations of enforced inactivity, such as hospitalisation or acute immobilisation. We have previously emphasised this aspect in the Introduction, and we have further clarified this point in the Discussion to better highlight the translational context and intended scope of the model. Lines 807-810.

Dear Dr Motanova,

Re: JP-RP-2026-291588R1 "**Preserved mitochondrial respiration in the presence of oxidative stress and reduced mitochondrial mass after 10-day bed rest in older adults**" by Evgeniia Motanova, Lucrezia Zuccarelli, Evgenii Lysenko, Stefano Amoretti, Marco Pirazzini, Ornella Rossetto, Lorenza Brocca, Roberto Bottinelli, Maria Antonietta Pellegrino, Giovanni Baldassarre, Clarissa Gissi, Giovanna Lippe, Mladen Gasparini, Bostjan Simunic, Rado Pisot, Bruno Grassi, and Marco Narici

We are pleased to tell you that your paper has been accepted for publication in The Journal of Physiology.
- Authors are required to fully disclose the use of any Generative AI (GAI) tools used in the preparation of their manuscript. This disclosure should also be included as a statement alongside the Additional Information and Data Availability statements towards the end of the article under the heading 'Generative AI Statement', and include the name(s), model and version of AI technology used, and exact details on the purpose for, and methods of, its use.

If no AI tool has been used, this should also be specifically stated within the manuscript.

The last Word (or similar) version of the manuscript provided will be used by the Production Editor to prepare your proof. When this is ready you will receive an email containing a link to Wiley's Online Proofing System. The proof should be thoroughly checked and corrected as promptly as possible.

Authors should note that it is too late at this point to offer corrections prior to proofreading. Major corrections at proof stage, such as changes to figures, will be referred to the Editors for approval before they can be incorporated. Only minor changes, such as to style and consistency, should be made at proof stage. Changes that need to be made after proof stage will usually require a formal correction notice.

All queries at proof stage should be sent to: TJP@wiley.com.

Following copyediting and typesetting the accepted version will be published online.

Yours sincerely,

Karyn Hamilton
Senior Editor
The Journal of Physiology

IMPORTANT POINTS TO NOTE FOLLOWING ACCEPTANCE OF YOUR PAPER:

• IMPORTANT NOTICE ABOUT OPEN ACCESS: To assist authors whose funding agencies mandate immediate public access to published research findings, The Journal of Physiology allows authors to pay an Open Access (OA) fee to have their papers made freely available immediately on publication.

The Corresponding Author will receive an email from Wiley with details on how to register or log in to Wiley Authors where you will be able to place an order.

• You can check if your funder or institution has a Wiley Open Access Account here:
https://authors.wiley.com/author-resources/Journal-Authors/open-access/author-compliance-tool.html

MEMBER DISCOUNT: Corresponding authors who are Members of the Physiological Society are eligible for a 20% discount on the cost of the Article Processing Charge (APC). To claim this discount, the Corresponding Author will need to log in to the Physiological Society Membership portal and retrieve the discount code (https://www.physoc.org/journals-and-media/journals/journal-discount-for-members/). This code should then be quoted in the billing section of Wiley Authors when selecting the open access option.

• TRANSPARENT PEER REVIEW POLICY: To improve the transparency of its peer review process, The Journal of Physiology publishes online as supporting information the peer review history of all articles accepted for publication. Readers will have access to decision letters, including Editors' comments and referee reports, for each version of the manuscript as well as any author responses to peer review comments. Referees can decide whether or not they wish to be named on the peer review history document.

• You can help your research get the attention it deserves! Check out Wiley's free Promotion Guide for best-practice recommendations for promoting your work at: www.wileyauthors.com/eeo/guide. You can learn more about Wiley Editing Services which offers professional video, design, and writing services to create shareable video abstracts, infographics,

conference posters, lay summaries, and research news stories for your research at: www.wileyauthors.com/eeo/promotion.

• If you would like to receive our 'Research Roundup', a monthly newsletter highlighting the cutting-edge research published in The Physiological Society's family of journals (The Journal of Physiology, Experimental Physiology, Physiological Reports, The Journal of Nutritional Physiology and The Journal of Precision Medicine: Health and Disease), please click this link, fill in your name and email address and select 'Research Roundup':
https://www.physoc.org/journals-and-media/membernews

EDITOR COMMENTS

Reviewing Editor:

Thank you for submitting your revised manuscript. The reviewer has indicated all prior concerns have now been addressed.

Senior Editor:

Thank you for submitting your revised manuscript. We are pleased to accept it for publication in The Journal of Physiology. Thank you for your interest in The Journal and Congratulations!

REFEREE COMMENTS

Referee #1:

You have addressed my comments well, and I have nothing further to add.
